# A Rapid and Sensitive LAMP Assay for the Detection of *Klebsiella aerogenes* in Food Matrices

**DOI:** 10.3390/foods15081277

**Published:** 2026-04-08

**Authors:** Mila Djisalov, Marija Pavlović, Ljiljana Janjušević, Ljiljana Šašić Zorić, Željko D. Popović, Ivana Gadjanski

**Affiliations:** 1 BioSense Institute, Center for Biosystems, University of Novi Sad, 21000 Novi Sad, Serbia; 2 Faculty of Sciences, University of Novi Sad, 21000 Novi Sad, Serbia; zeljko.popovic@dbe.uns.ac.rs; 3 Institute for Oncology and Radiology of Serbia, Department of Experimental Oncology, 11000 Belgrade, Serbia

**Keywords:** LAMP, Klebsiella aerogenes, molecular diagnostics, on-site detection, *HDC* gene

## Abstract

Foodborne pathogens such as *Klebsiella aerogenes* pose a threat to food safety, highlighting the need for rapid, reliable detection methods amid rising contamination risks in production chains. In this study, a loop-mediated isothermal amplification (LAMP) assay was developed and validated to detect the histidine decarboxylase (*HDC*) gene of *K. aerogenes*. The assay was optimized for specificity and sensitivity, tested on pure bacterial genomic DNA and artificially contaminated food matrices (vegetables and meats), and evaluated against real-time PCR (qPCR). To evaluate performance under different DNA quality conditions and simulate laboratory versus on-site workflows, two extraction approaches were compared: a standard laboratory protocol yielding high-purity DNA and a crude extraction method producing low-purity DNA, mimicking the presence of inhibitors commonly encountered in routine analysis and enabling practical on-site detection where commercial kits are not feasible. The developed LAMP assay achieved maximum specificity with no cross-reactivity to related species, limits of detection of 240 fg/reaction for pure bacterial DNA and 0.4 pg/µL in *K. aerogenes* artificially contaminated food samples, and a reaction time under 30 min—outperforming real-time PCR in speed and robustness. This cost-effective method provides a scalable tool for near-real-time monitoring of *K. aerogenes* in food production, enhancing safety and enabling early outbreak detection.

## 1. Introduction

*Klebsiella* spp. has been widely recognized as an important and problematic foodborne pathogen since the 1970s [1,2,3,4]. Despite decades of research and improvements in food safety practices, *Klebsiella* species remain a safety challenge, driven by the emergence of multidrug-resistant and hypervirulent strains that complicate detection and control efforts [5,6]. Most studies focus on the species *K. pneumoniae* due to its documented high clinical and epidemiological relevance [7,8], while often neglecting *K. aerogenes*, a species recognized by the World Health Organization (WHO) as a critical-priority pathogen within the ESKAPE group due to the rise of antibiotic-resistant strains [9]. *Klebsiella aerogenes*, formerly known as *Enterobacter aerogenes*, is a Gram-negative bacterium from the family *Enterobacteriaceae.* It has the ability to survive in diverse environments, contaminate a wide range of foods, and exhibit intrinsic and acquired antimicrobial resistance [2,10]. As a result, *K. aerogenes* remains a relevant and unresolved issue in contemporary food microbiology and public health. *Klebsiella pneumoniae* and *Klebsiella aerogenes* have been classified among the secondary bacterial causes associated with foodborne illness contexts, suggesting that while they may not be leading epidemiological agents, they are not negligible in food-associated transmission scenarios [11,12].

Moreover, *Klebsiella* spp. are increasingly recognized in the context of the “One Health” approach [13]. Environmental and food-chain transmission of *Klebsiella* spp. has been extensively explored. For instance, *K. pneumoniae* was isolated from raw lettuce, and some of the isolates were multidrug resistant, indicating that poor hygiene, contaminated irrigation water, and inadequate post-harvest handling can facilitate its transmission to consumers [14]. In addition, *Klebsiella* spp. and related *Enterobacteriaceae*, including *K. aerogenes*, have been recovered from fresh vegetables and other marketed agricultural foodstuffs, where antibiotic-resistant isolates further underscore their relevance as food-chain–associated hazards [15]. For example, in 2013, *K. aerogenes* was successfully isolated from fresh vegetables sourced from various locations in Valencia [15]. Furthermore, a more recent investigation conducted in Bangladesh focused on detecting *K. aerogenes* in raw milk revealed that 36.44% of the tested milk samples were positive for *Klebsiella* species, and the isolates were identified as closely related to *K. aerogenes* [16]. Moreover, a genomic analysis of various food samples, including leafy greens and meat, revealed the presence of *K. aerogenes* alongside other *Klebsiella* species [17]. In 2023, Melebari provided evidence of *K. aerogenes* contamination in restaurant environments [18], and in 2025, Parivendhan et al. (2024) [19] isolated *K. aerogenes* from street food, further demonstrating its role as a foodborne pathogen in retail and ready-to-eat food settings. These findings underscore the role of *K. aerogenes* within the broader foodborne bacterial community and highlight its relevance to food safety and public health. Therefore, a comprehensive pathogen detection strategy is necessary to adapt food safety policies to address the emerging dimension of bacterial transmission and mitigate the risks posed by foodborne pathogens, including *Klebsiella* spp., to human health.

Considering the frequency of contamination and importance of *Klebsiella* spp. as foodborne pathogens, there is a critical need for standardized detection methods across food matrices that will enable rigorous monitoring of foodborne infections and risk assessments. Moreover, in the context of the global food industry, there are clear advantages to implementing precise, rapid, and user-friendly detection methods that can identify pathogenic bacteria directly within food matrices and along the food production chain. Although molecular detection techniques based on isothermal amplification, particularly loop-mediated isothermal amplification (LAMP), have demonstrated strong potential for rapid, sensitive, and robust detection of closely related species such as *K. pneumoniae* in food and clinical samples [20], there is a notable lack of published LAMP assays explicitly targeting *K. aerogenes* in food matrices. This gap persists despite repeated reports documenting the presence of *K. aerogenes* in food production environments and in retail food products, highlighting a methodological shortcoming in current food microbiology surveillance tools.

With the aim of facilitating early detection and enabling transmission monitoring of *K. aerogenes*, a novel LAMP assay targeting the *K. aerogenes HDC* gene was developed and optimized for rapid, specific, and robust on-site detection in food samples.

## 2. Materials and Methods

**Figure 1:**
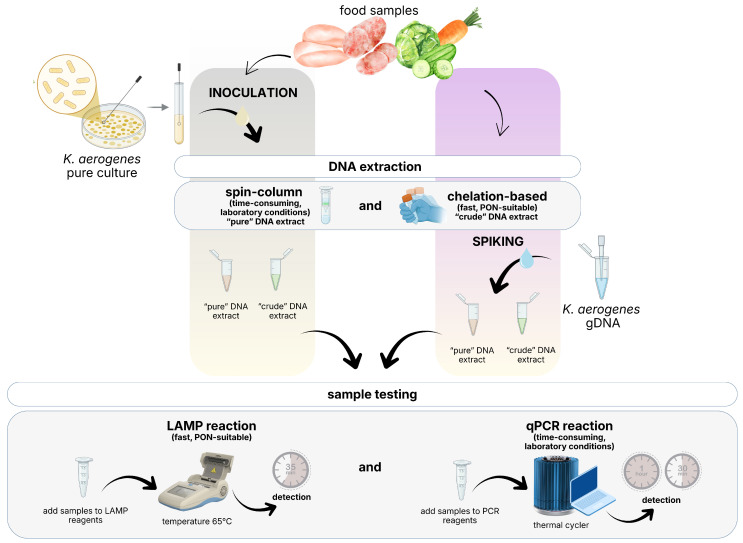
Design and validation of the LAMP assay for on-site, i.e., Point-of-Need (PON) detection of *Klebsiella aerogenes* in food: graphical workflow overview. Created by the authors using Canva (Canva Pty Ltd., Sydney, Australia, www.canva.com).

The graphical overview (Figure 1) illustrates the workflow for design and validation of the LAMP assay for on-site detection of *K. aerogenes* in food samples.

### 2.1. Bacterial Strains and Culture Conditions

The bacterial strains used in this study are listed in Table S1. A day before the DNA extraction step, all bacterial species were cultivated on Tryptone Soya Agar (TSA) (Millipore, Burlington, MA, USA) at 37 °C for 24 h (overnight) in a thermostat incubator (Incubator IN55, Memmert, Schwabach, Germany).

### 2.2. Food Samples

Food samples used for validation of the developed LAMP assay included vegetable samples (cucumber—*Cucumis sativus* L., lettuce—*Lactuca sativa* L., and carrot—*Daucus carota* L.), and meat samples (fresh chicken breasts and chicken salami (a commercially available, ready-to-eat, cooked poultry sausage product)). All food samples were purchased from local markets in Novi Sad, Serbia, and transported to the laboratory of the BioSense Institute (Novi Sad), where they were processed under aseptic conditions. To evaluate bacterial contamination, vegetable samples were washed with 3 mL of PCR-grade water and 1 mL of the wash water was transferred onto Tryptone Soya Agar (TSA) plates. Plates were incubated for 24 h at 37 °C. For meat samples, standard bacterial pre-enrichment was performed by incubating 250 mg of each meat sample (chicken breasts and salami) in 2 mL of Tryptone Soya Broth (TSB) (Millipore, Burlington, MA, USA) for 24 h at 37 °C to mimic conventional microbiological workflows for tissue-embedded bacteria following the ISO 6579-1 principle [21]. Following incubation in TSB, samples were spread-plated onto TSA plates and incubated for 24 h at 37 °C to obtain pure colonies. These matrix-specific protocols reflect real-world contamination patterns: surface recovery from vegetables vs. internal bacterial growth within the meat matrix.

For DNA extraction (LAMP confirmation), all colonies grown on TSA plates were scraped off and pooled into 2 mL Eppendorf tubes for the extraction process. For biochemical identification, individual colonies from the same TSA plates were subcultured onto fresh TSA plates, incubated for 24 h at 37 °C, and then tested.

#### Targeted, Controlled Contamination of Food Samples

The absence of *K. aerogenes* in analysed food samples prior to artificial inoculation was confirmed using the developed real-time LAMP assay and standard biochemical identification tests (Gram staining, morphology, nitrate reductase, catalase, oxidase, indole, Voges-Proskauer, and methyl-red tests) [22] on pre-enrichment food cultures, as described in Section 2.2. Targeted, controlled contamination of food samples (vegetables and meat) was conducted using two approaches: (1) spiking food DNA with *K. aerogenes* genomic DNA (gDNA) and (2) food samples inoculation with *K. aerogenes* bacterial suspension. In the first approach, genomic DNA from *K. aerogenes* (at a concentration of 59.9 ng/μL) was directly added to tubes containing food (vegetable and meat) DNA extracts, achieving a final concentration of 1 ng/μL of *K. aerogenes* gDNA in the spiked food DNA sample, which was then used for LAMP analysis. In the second approach, *Klebsiella aerogenes* bacterial suspension for direct food contamination was prepared and adjusted to a concentration of 1.5 × 10^9^ CFU/mL (5 McFarland Standard, McF) using a DEN-1 densitometer (BioSan, Riga, Latvia). For vegetable samples, 100 μL of the bacterial suspension was added to tubes containing 125 mg of vegetable material. Following inoculation, 900 μL of extraction buffer was added (to reach a total volume of 1 mL and a final concentration of ~10^8^ CFU/mL of *K. aerogenes*), after which, the samples underwent DNA extraction and subsequent LAMP analysis. For meat samples, 100 μL of the bacterial suspension was added to tubes containing 250 mg of each meat sample and 1650 μL of Tryptone Soya Broth (TSB). The tubes were incubated at 37 °C for 24 h, after which, the meat samples were subjected to DNA extraction. For inoculated samples, concentrations of DNA extracts were normalized to 1 ng/μL total DNA prior to LAMP analysis.

### 2.3. DNA Extraction

#### 2.3.1. DNA Extraction from Bacterial Cultures

Bacterial DNA was extracted using the GeneJET Genomic DNA Purification Kit (Thermo Scientific™, Waltham, MA, USA). For Gram-positive and Gram-negative bacterial cultures, the respective protocols provided by the manufacturer were followed. DNA concentration and purity were measured using a BioSpec-nano spectrophotometer (Shimadzu, Kyoto, Japan). The extracted DNA samples were stored at −20 °C until further use.

#### 2.3.2. DNA Extraction from Vegetables

DNA from vegetable samples (carrot, cucumber, and lettuce) was extracted using two methods: the Chelex 100 method, suitable for in-field applications, and the Plant/Fungi DNA Isolation Kit (Norgen Biotek, Thorold, ON, Canada) following manufacturer’s instructions. The Chelex 100 method employed a crude extraction protocol that combines the Chelex reagent with an alkaline PEG lysis buffer, whose composition follows the formulation described by Tomlinson and Boonham [23]. For this method, 125 mg of each vegetable sample, including both non-processed and those subjected to targeted, controlled contamination, were placed in a 5 mL microcentrifuge tube containing 0.4 g of Chelex 100 resin (Bio-Rad, Hercules, CA, USA), one sterilized metal bead (10 mm in diameter), and 900 µL of alkaline PEG lysis buffer pH 13.3–13.5 (60% of polyethylene glycol 200 (PEG 200) (Sigma-Aldrich Co. LLC, Darmstadt, Germany), 20 mM NaOH (Sigma-Aldrich, St. Louis, MO, USA)). The tube was shaken vigorously for 1 min to mechanically disrupt the tissue, followed by incubation at room temperature for 20 min to facilitate DNA release and allow the Chelex resin to settle and remove impurities. After incubation, a 20 µL aliquot of the supernatant was diluted 1:50 with 1 mL of dilution buffer (1× TE buffer, pH 8) (Sigma-Aldrich, St. Louis, MO, USA). This diluted DNA extract was used for LAMP analysis. DNA concentration and purity were measured using a BioSpec-nano spectrophotometer (Shimadzu, Kyoto, Japan). The extracted DNA samples were stored at −20 °C until further use.

#### 2.3.3. DNA Extraction from Meat and a Meat Product

DNA from meat samples (fresh chicken breasts and chicken salami) was extracted using two methods: the aforementioned Chelex 100 method, suitable for in-field applications, and the protocol described in the DNeasy PowerFood Microbial Kit (Qiagen, Hilden, Germany). Within this study, the Chelex 100 method, originally applied to vegetable samples, was adapted and validated for meat samples by incorporating a meat-maceration step. Briefly, 250 mg of each meat sample, both non-processed and those subjected to controlled contamination, was placed into sterile 2 mL tubes. Next, 750 µL of 1× PBS was added to the sample. The samples were macerated using a sterile tube homogenizer for approximately 2 min until a homogeneous mixture was achieved. The tube was then left for 10 min to allow larger particles to settle, followed by processing according to the Chelex 100 protocol described for vegetables. For the DNeasy PowerFood Microbial Kit method, 250 mg of each meat sample (non-processed and contamination-subjected) was used as starting material. DNA extraction and purification were performed according to the manufacturer’s instructions. DNA concentration and purity were measured using a BioSpec-nano spectrophotometer (Shimadzu, Kyoto, Japan). The extracted DNA samples were stored at −20 °C until further use.

### 2.4. LAMP Assay for K. aerogenes Detection

#### 2.4.1. LAMP Primer Design

The histidine decarboxylase coding gene (*HDC*) of *K. aerogenes* (GenBank accession No. NZ_CP041925, gene tag FPV33_RS04375) was selected as the target gene for primer design, and its highly conserved sequences were identified using the Nucleotide Basic Local Alignment Search Tool (Nucleotide BLAST; NCBI BLAST web interface, https://blast.ncbi.nlm.nih.gov/Blast.cgi, accessed on 10 January 2023, latest version available at that date). BLAST analysis was performed using the megablast algorithm with default parameters: Expect threshold of 0.05, 100 Max target sequences, Standard nr/nt databases. The LAMP primers were designed using Primer Explorer V5 software (Eiken Chemical Co., Ltd., available online at https://primerexplorer.eiken.co.jp/lampv5e/index.html, accessed on 10 January 2023, latest version available at that date) targeting different sequences within specific regions of the *HDC* gene. To minimize the risk of undesired secondary structures, such as hairpins, homodimers, and heterodimers, the primer sequences were analysed using the OligoAnalyzer software provided by Integrated DNA Technologies (IDT) (available at https://eu.idtdna.com/pages/tools/oligoanalyzer, accessed on 10 January 2023, latest version available at that date). Designed LAMP primer sequences and locations on the *HDC* sequence are shown in Supplementary Data (Table S2 and Figures S1–S3). Following primer design and preliminary evaluation, primer candidate set PC2 was selected for further analysis. The specificity of PC2 was subsequently evaluated in silico. Five *HDC* gene sequences of *K. aerogenes* were retrieved from the NCBI database (https://www.ncbi.nlm.nih.gov/, accessed on 12 January 2023) and aligned with the PC2 primer sequences to confirm full complementarity within the target regions (Table S3, Supplementary Data S1).

#### 2.4.2. LAMP Assay Optimization

In order to optimize the LAMP reaction, we evaluated the efficiency of three primer candidate (PC) sets targeting the *K. aerogenes HDC* gene (Table S2) and their reaction times. The LAMP reactions were prepared according to the protocols outlined in the WarmStart LAMP Kit and WarmStart^®^ Colorimetric LAMP Kit (New England Biolabs, Ipswich, MA, USA). To optimize the amplification, different reaction times (30, 45, and 60 min) were tested at a constant temperature of 65 °C. All assays were performed in triplicate. Amplification was carried out using a Genie^®^ III Instrument (OptiGene, Horsham, UK) for real-time LAMP and a Thermo-Shaker TS-100C (BioSan, Riga, Latvia) for colorimetric LAMP, both at 65 °C.

#### 2.4.3. LAMP Assay Specificity

The specificity of the best-performing LAMP primer set (PC2) was tested using gDNA from five foodborne pathogenic bacterial strains: two Gram-positive (*Staphylococcus aureus* and *Bacillus subtilis*) and three Gram-negative (*Alcaligenes faecalis*, *Salmonella enterica*, and *Escherichia coli*). LAMP reactions were performed using both colorimetric and real-time approaches at 65 °C for 30 min (followed by an additional 10 min at 85 °C to terminate the reaction for colorimetric LAMP). All assays were conducted in triplicate. Amplification products were analyzed by 2% agarose gel electrophoresis to confirm specific amplification. In addition to experimental validation, the specificity of the PC2 LAMP primers was further evaluated in silico. To assess potential cross-reactivity with closely related *Enterobacteriaceae*, *HDC* sequences from *Klebsiella pneumoniae*, *Morganella morganii*, *Citrobacter youngae*, *Raoultella ornithinolytica*, and *Raoultella planticola* (Table S4) were aligned with the corresponding *HDC* sequences from *K. aerogenes* (Supplementary Data S2). These species were selected because they are phylogenetically related to *K. aerogenes* and may harbor homologous *HDC* genes [24] and therefore represent potential sources of false-positive amplification in LAMP assays.

#### 2.4.4. LAMP Assay Sensitivity

The limit of detection (LoD) for the PC2 LAMP primer set was determined using 10-fold serial dilutions of purified *K. aerogenes* gDNA (stock concentration: 59.9 ng/µL) prepared in molecular-grade water, ranging from 5.99 ng/µL to 5.99 pg/µL. Both colorimetric and real-time LAMP assays were performed at 65 °C for 30 min, with all reactions conducted in triplicate. The LoD was defined as the lowest template concentration at which all technical replicates showed positive amplification, confirmed by visual color change (colorimetric), amplification curves (real-time), and 2% agarose gel electrophoresis. For the real-time LAMP assays, time-to-threshold (Tt) values, which represent the time required for the fluorescent intensity to reach or exceed a defined reaction threshold, were recorded [25]. Tt values were used as an additional criterion to determine the lowest detectable concentration; all replicates showed positive amplification when Tt was ≤30 min. The threshold was automatically set by the Genie^®^ III instrument for each reaction well based on baseline fluorescence and dynamic range (Genie^®^ III User Manual, Instrument Software Version v3.18.2), typically corresponding to 10–20% of maximum fluorescence change.

### 2.5. Validation of LAMP Assay in Food Matrices

DNA extracts from inoculated food and spiked food DNA (prepared as described in Section Targeted, Controlled Contamination of Food Samples) were analyzed by real-time LAMP using the PC2 primer set. The limit of detection (LoD) was determined using 10-fold serial dilutions (1:10 to 1:10,000) of food DNA. All reactions were performed in triplicate, except for meat-derived samples, which were analyzed in duplicate (due to limited sample availability). Amplification was monitored using Tt values, which were analyzed by one-way ANOVA followed by Tukey’s multiple comparisons test (*p* < 0.05) using GraphPad Prism 8 (GraphPad Software, Boston, MA, USA). Non-spiked food DNA and DNA extracts from non-inoculated food served as negative controls.

### 2.6. Real-Time PCR Analysis

F3 and B3 primers from the PC2 LAMP primer set were used for real-time PCR. Real-time PCR was performed using the MyGo Mini S instrument (Azura Genomics Inc., Raynham, MA, USA) with Maxima SYBR Green/ROX qPCR Master Mix (2X) (Thermo Scientific™, Waltham, MA, USA). The same inoculated food DNA extracts (as described in Section Targeted, Controlled Contamination of Food Samples) were analyzed in 15-μL reactions composed of 7.5 µL of 2X master mix, 0.45 µL each of 10 µM F3/B3 primers, 5.0 µL of DNA template, and 1.6 µL of PCR-grade water. Thermal cycling conditions were: initial denaturation at 95 °C for 10 min, followed by 30 cycles of 95 °C (30 s), 56 °C (30 s), and 72 °C (30 s), with high-resolution melting from 65 °C to 95 °C (1.5 °C/s initial, 0.05 °C/s final). Control samples were *K. aerogenes* gDNA (positive control) and PCR-grade water (no-template control—NTC). All reactions were performed in triplicate.

## 3. Results and Discussion

### 3.1. Design and Validation of LAMP Primers

BLAST analysis of the *K. aerogenes HDC* sequence (NZ_CP041925, FPV33_RS04375) against 76 NCBI *K. aerogenes HDC* sequences confirmed high conservation (100% query coverage, 97.83–100% identity). Three LAMP primer sets were then designed targeting these conserved regions (Table S2) and screened for specificity by BLAST. Absence of self-dimers/heterodimers was validated using OligoAnalyzer Tool (Integrated DNA Technologies, Coralville, IA, USA). Sequence alignment of five representative *K. aerogenes* strains (Supplementary Data S1) confirmed full complementarity of the PC2 primer set within target regions. In silico specificity analysis (Table S4, Supplementary Data S2) showed low PC2 sequence complementarity with *HDC*-positive *Enterobacteriaceae* (*K. pneumoniae*, *M. morganii*, *Raoultella* spp. and *C. youngae*), establishing PC2 species-specificity for *K. aerogenes.*

**Figure 2:**
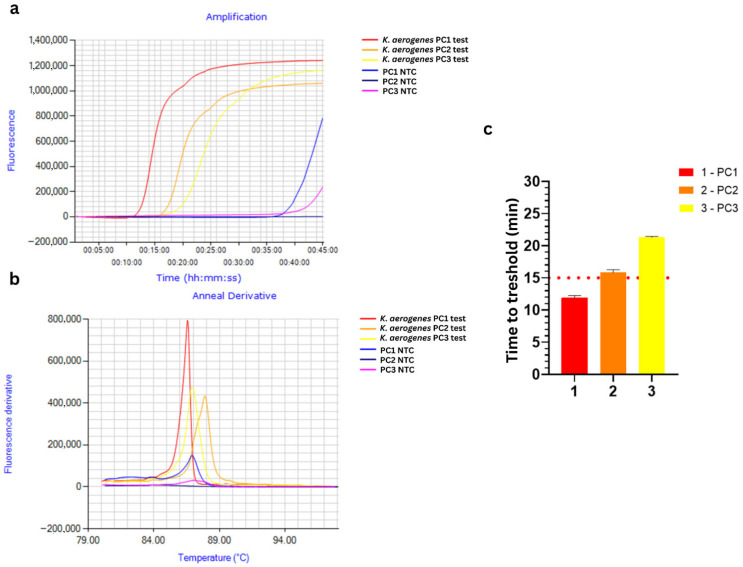
Real-time LAMP assay validation of PC1, PC2, and PC3 primer candidates for *Klebsiella aerogenes* detection (65 °C, 45 min): (**a**) Amplification curves of *K. aerogenes* gDNA using PC1 (red), PC2 (orange), and PC3 (yellow) primer candidate sets; (**b**) Melting curves of amplification products; (**c**) time to threshold (Tt) values for each primer candidate set. PCR-grade water served as NTC for all primer candidate sets (PC1 NTC—light blue, PC2 NTC—blue, PC3 NTC—pink).

Performance of three designed LAMP primer sets (PC1–PC3, Table S2) was validated using *K. aerogenes* gDNA as a positive control and PCR-grade water as a no-template control (NTC). Initial 60 min reaction (65 °C) revealed late amplification in all NTCs (PC1 ~ 30 min, PC2/PC3 ~ 45 min) (Figure S4), reflecting the intrinsic property of LAMP reactions to produce nonspecific signals beyond optimized time [26,27,28,29]. Therefore, primer sets were validated in 45 min reaction, where PC2 showed amplification at Tt ~ 16 min with no NTC signal (orange curve in Figure 2a), while PC1 (Tt ~ 12 min) (red curve in Figure 2a) produced false positives (blue curve in Figure 2a) and PC3 (Tt ~ 21 min) (yellow curve in Figure 2) showed late NTC amplification after 38 min (pink curve in Figure 2a). Based on these results, PC2 was selected for further optimization. The final reaction time was set to 30 min, eliminating the possibility of late NTC signals while maintaining rapid PC2 detection.

**Figure 3:**
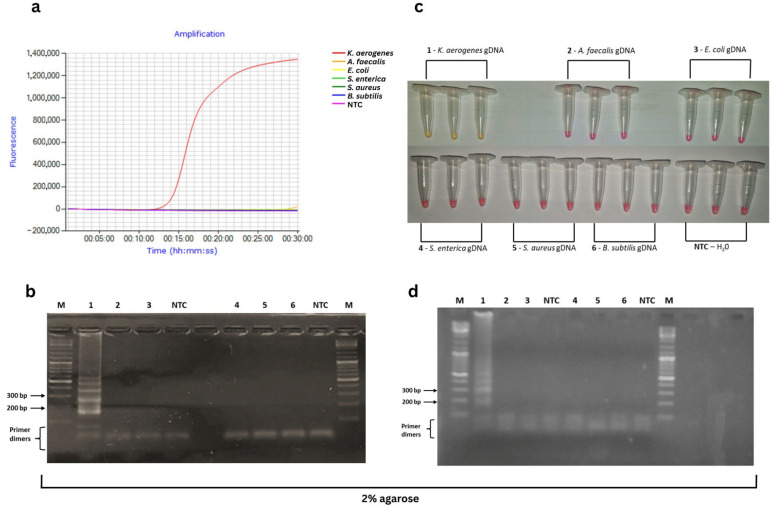
Specificity validation of PC2 LAMP primers for *Klebsiella aerogenes* detection: (**a**) Real-time LAMP curves (30 min, 65 °C): *K. aerogenes* gDNA (red curve, positive), non-specific gDNA (*Alcaligenes faecalis*, orange; *Escherichia coli*, yellow; *Salmonella enterica*, light green; *Staphylococcus aureus*, dark green; *Bacillus subtilis*, blue), NTC (pink); (**b**) Agarose gel electrophoresis of real-time LAMP products: M—marker, 1—*K. aerogenes*; 2—*A. faecalis*; 3—*E. coli*, 4—*S. enterica*; 5—*S. aureus*, 6—*B. subtilis*; NTC—no-template control; (**c**) Colorimetric LAMP results: yellow (*K. aerogenes*), pink (all non-specific/NTC); (**d**) Agarose gel electrophoresis of colorimetric LAMP products: M—marker, 1—*K. aerogenes*; 2—*A. faecalis*; 3—*E. coli*, 4—*S. enterica*; 5—*S. aureus*, 6—*B. subtilis*; NTC—no-template control.

In silico specificity confirmed perfect complementarity of PC2 primers with five *K. aerogenes HDC* sequences available in GenBank (Table S3, Supplementary Data S1), while *HDC* sequences from closely related *Enterobacteriaceae* (*K. pneumoniae*, *M. morganii*, *C. youngae*, *R. ornithinolytica*, *R. planticola*) showed substantial sequence divergence comparing to the *K. aerogenes HDC* target sequence used for PC2 primer design (Table S4, Supplementary Data S2). The specificity of the PC2 primer set was experimentally confirmed using gDNA from *K. aerogenes* and five non-target foodborne bacteria (*S. aureus*, *B. subtilis*, *A. faecalis*, *S. enterica*, *E. coli*). Real-time LAMP showed amplification only for *K. aerogenes* (Tt ~ 16 min, red curve, Figure 3a), with characteristic sigmoidal curve. No amplification was detected in non-target species (Figure 3a,b). No outliers or inconsistent amplification patterns were observed during the experiments. Colorimetric LAMP confirmed specificity, with color change from pink to yellow observed exclusively in *K. aerogenes* samples after 30 min (Figure 3c). Agarose gel electrophoresis only showed specific bands for *K. aerogenes* (Figure 3b,d), with no non-specific products in negative controls.

**Figure 4:**
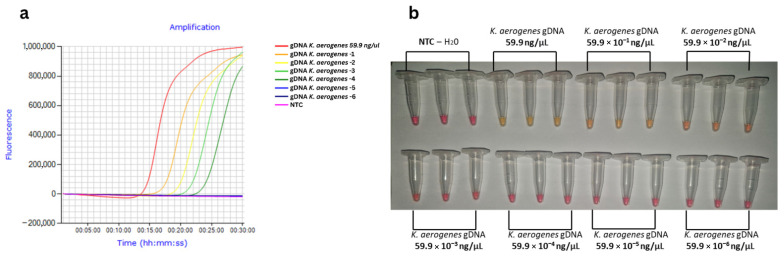
Limit of detection (LoD) estimation for PC2 LAMP primers targeting *Klebsiella aerogenes* gDNA (10-fold serial dilutions): (**a**) Real-time LAMP curves (LoD: 10^−4^ dilution, dark green curve, 240 fg/reaction); (**b**) Colorimetric LAMP results (LoD: 10^−2^ dilution, 24 pg/reaction; yellow = positive, pink = negative).

The limit of detection (LoD) of the LAMP assay was determined using the best-performing primer set (PC2), and a 10-fold serial dilution of *K. aerogenes* gDNA. Real-time LAMP’s LoD occurred at 10^−4^ dilution (240 fg/reaction in 25 µL reaction volume), with regular Tt intervals and a linear range across all tested concentrations (R^2^ = 0.9923) (dark green curve in Figure 4a; detailed linear regression and CV% < 5% for triplicate Tt values in Supplementary Data S3). No outliers or inconsistent amplification patterns were observed during the experiments. For colorimetric LAMP, the determined LoD was the 10^−2^ dilution (24 pg/reaction) (Figure 4b), thus being less sensitive than fluorescent quantification (real-time LAMP) due to the naked-eye detection. Superior sensitivity of real-time LAMP reflects the higher efficiency of the fluorescent dye compared to colorimetric pH-sensitive dyes, while precise quantification outperforms naked-eye reading [30,31]. These LoDs are comparable to reported LoDs of *K. pneumoniae* LAMP assays (115 fg/µL [32]; 100 fg/µL fluorometric [33]), establishing PC2 as the first primer set reported for highly sensitive detection of *K. aerogenes* using the LAMP method.

### 3.2. Testing Food Samples for K. aerogenes Contamination

Real-time LAMP assays on gDNA from pre-enrichment cultures and direct DNA extracts of all food samples showed amplification exclusively in positive controls (red curves in Figure S5a,b), confirming the complete absence of natural *K. aerogenes* contamination. Biochemical identification tests further corroborated these findings, showing biochemical profiles inconsistent with *K. aerogenes* reference characteristics (Voges-Proskauer-positive, methyl red-negative, nitrate reduction-positive, catalase-positive, oxidase-negative, indole-negative) across all food matrix samples (Table S5), establishing a reliable contamination-free baseline for further validation of the developed LAMP assay on DNA extracts of artificially inoculated food samples and spiked food DNA.

### 3.3. LAMP Assay on Spiked Food DNA Samples

**Figure 5:**
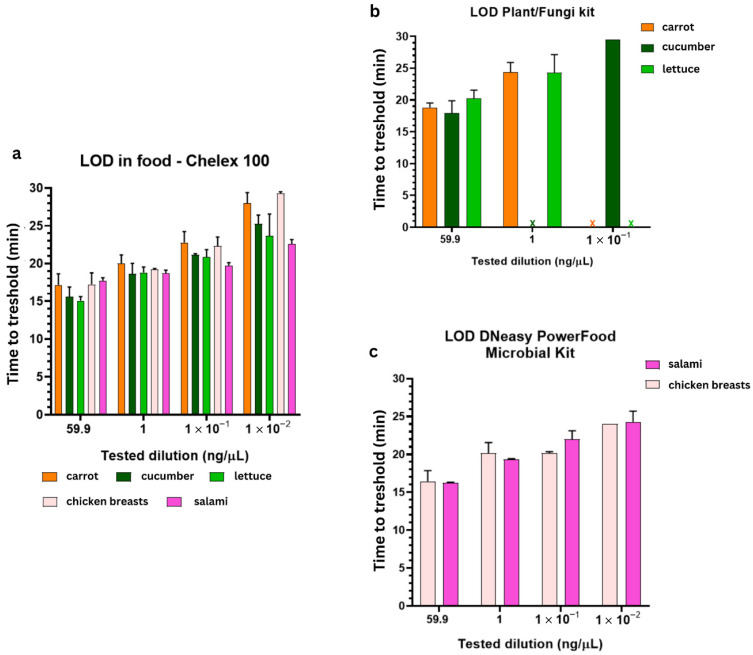
Estimation of *Klebsiella aerogenes* LAMP assay LoD in spiked food DNA extracts from vegetables and meat extracted using different DNA extraction methods: (**a**) Chelex 100 extraction; (**b**) Plant/Fungi DNA Isolation Kit; (**c**) DNeasy PowerFood Microbial Kit. Tested samples: 59.9 ng/µL *K. aerogenes* pure gDNA (used as a positive control), spiked food DNA samples containing 1 ng/µL, 10^−1^ ng/µL and 10^−2^ ng/µL *K. aerogenes* gDNA.

The limit of detection (LoD) for *K. aerogenes* gDNA in spiked food DNA extracts from vegetables (carrot, cucumber, lettuce) and meat (chicken breast, salami) was compared between different DNA extraction methods. The achieved LoD of real-time LAMP after Chelex 100 extraction was 0.4 pg/µL across all samples (Figure 5a and Figure S6), with Tt values of 23–28 min (Figure 5a, Table S6), with the exception of chicken breast DNA (>29 min, *p* < 0.05 vs. lettuce/salami). On the other hand, the LoD of real-time LAMP for food DNA samples extracted using Plant/Fungi DNA Isolation Kit was 40 pg/µL for carrot and lettuce, but no detection was achieved for cucumber (Figure 5b and Figure S7, Table S7). The LoD of real-time LAMP reaction for the meat DNA samples extracted using the DNeasy PowerFood Microbial Kit matched with LoD for Chelex DNA extracts—0.4 pg/µL (Figure 5c and Figure S8, Table S8; Tt 23–25 min).

As no prior LAMP assays exist for *K. aerogenes* in food, the comparison of achieved LoDs can be made vs. reported LAMP LoD for *Salmonella enteritidis* in spiked tomatoes (5 pg/µL [34], which showed that our assay, when coupled with Chelex based DNA extraction, is superior for pathogen detection in plant-based food samples. Chelex 100 DNA extraction has been proven superior to commercial kit for vegetable DNA extraction due to the use of robust, field-compatible homogenization combined with high tolerance to plant-derived PCR inhibitors such as polysaccharides, polyphenols, and tannins that inhibit LAMP enzymes). Additionally, we noticed that plant material type also affects Tt, with the faster Tt achieved in lettuce samples compared to cucumber samples. This likely reflects easier lysis of lettuce leaves versus the fibrous/waxy cucumber tissue, which contains ~90% carbohydrates in its cell walls [11,35]. In contrast, the LAMP results for meat samples were not dependent on the method of extraction or the type of meat sample.

### 3.4. LAMP vs. Real-Time PCR Detection in K. aerogenes-Inoculated Food Samples

**Figure 6:**
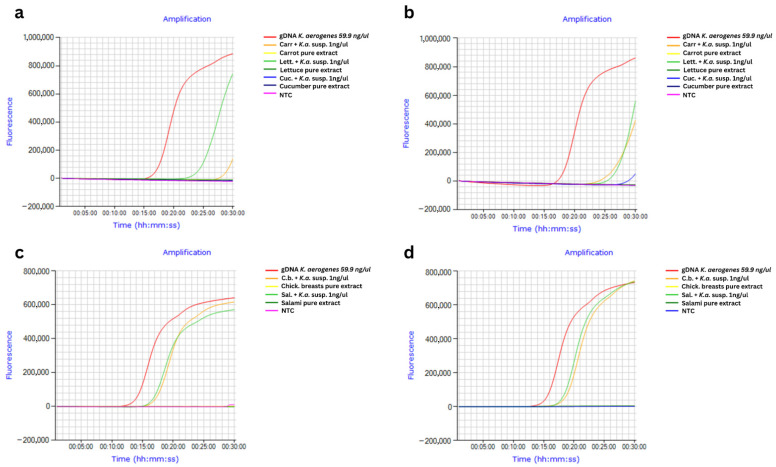
Real-time LAMP curves comparing *Klebsiella aerogenes* detection in DNA samples extracted from food using Chelex 100 vs. commercial kits. LAMP curves for DNA samples from vegetables: (**a**) Chelex 100 extraction; (**b**) Plant/Fungi DNA Isolation Kit. LAMP curves for DNA samples from meat: (**c**) Chelex 100 extraction; (**d**) DNeasy PowerFood Microbial Kit.

**Figure 7:**
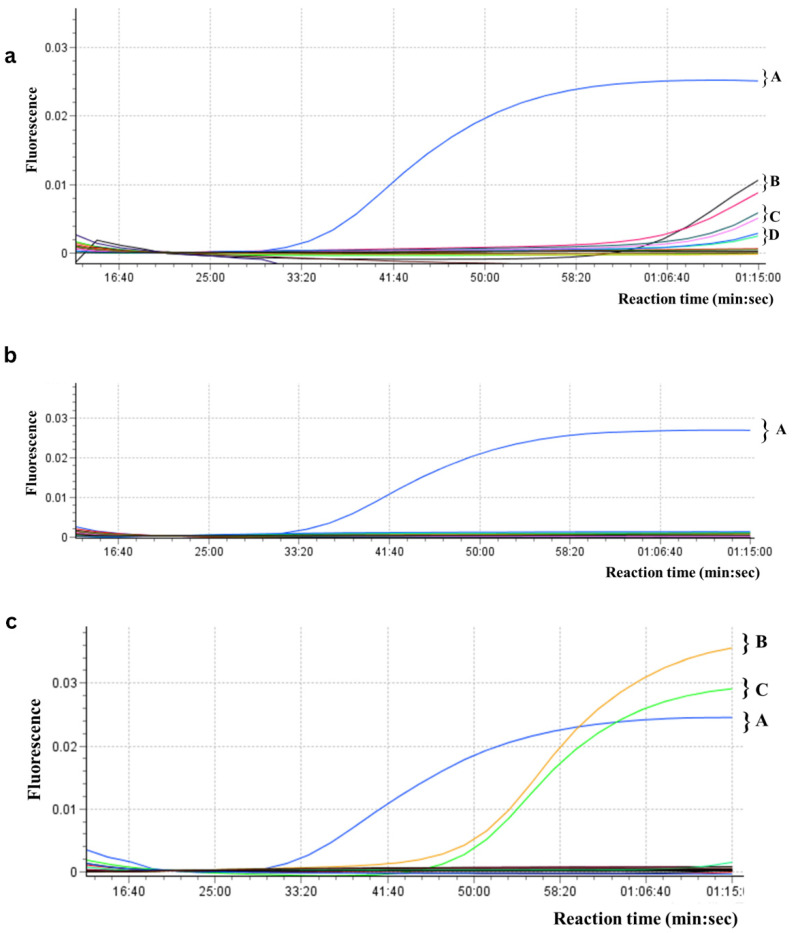
Real-time PCR amplification curves comparing *Klebsiella aerogenes* detection in DNA samples extracted from food using Chelex 100 vs. commercial kits. PCR amplification curves for DNA samples from vegetables: (**a**) Chelex 100 extraction: *K. aerogenes* gDNA positive control (blue curve, A), inoculated carrot extract (black/red curves, B), inoculated lettuce extract (dark green/pink curves, C), inoculated cucumber extract (dark blue/turquoise curves, D) technical replicates; (**b**) Plant/Fungi DNA Isolation Kit: *K. aerogenes* gDNA positive control (blue curve, A); inoculated carrot, lettuce and cucumber extracts (1 ng/µL) not amplified; negative controls not amplified. PCR amplification curves for DNA samples from meat: (**c**) Chelex 100 extraction and DNeasy PowerFood Microbial Kit: *K. aerogenes* gDNA positive control (blue curve, C), DNeasy Kit—inoculated salami extract (orange curve, A), inoculated chicken breast extract (green curve, B); Chelex 100—inoculated salami and chicken breast extracts (1 ng/µL) not amplified; negative controls not amplified.

Real-time LAMP assays successfully detected *K. aerogenes* gDNA in DNA extracts of inoculated food samples (vegetables (carrot, cucumber, lettuce) and meat (chicken breast, salami)) isolated using either the Chelex 100 method (Figure 6a,c; Tt 16–29 min for positives) or commercial kits (Plant/Fungi DNA Isolation Kit: Figure 6b, Tt 22–27 min; DNeasy PowerFood Microbial Kit: Figure 6d, Tt 17–18 min). The melting peaks matched those of the positive controls, and no amplification was observed in non-inoculated controls. Real-time PCR yielded delayed results compared to real-time LAMP, taking over than 1 h longer for vegetable DNA extracts isolated using the Chelex-based method (Figure 7a), and around 45 min longer for meat DNA extracts isolated using a commercial kit (Figure 7c). No results were obtained for vegetable DNA extracts isolated using a commercial kit (Figure 7b).

Based on these results, real-time LAMP was 2–3× faster than real-time PCR. This superior performance stems from Bst polymerase’s higher tolerance to inhibitors and LAMP’s use of high Mg^2+^ concentrations (6–8 mM) that neutralize contaminants, unlike Taq polymerase which requires purified DNA from complex food matrices [12,36].

## 4. Conclusions

We successfully developed and demonstrated a proof-of-concept study for a highly sensitive and specific LAMP assay for the rapid detection of *K. aerogenes* contamination in food samples. The optimized LAMP primer set (PC2) demonstrated excellent specificity for the *K. aerogenes HDC* gene, with no cross-reactivity observed in closely related bacterial species. The developed LAMP assay has a low LoD of 240 fg/reaction (10^−4^ dilution) for pure gDNA, and 0.4 pg/µL in spiked food DNA samples extracted using Chelex 100 method, showcasing its sensitivity in complex matrices. It provides a rapid, simple, and cost-effective method for detecting *K. aerogenes* contamination in food, with results available in under 30 min. In terms of field applicability, the assay’s minimal equipment requirements, short turnaround time, and robustness in spiked food matrices make it highly suitable for decentralized diagnostics, such as on-site testing in food processing plants or resource-limited labs. However, limitations include potential matrix-specific inhibition which may require optimization of the Chelex extraction or alternative pre-treatments to maintain LoD. Sample preparation thus remains a key factor influencing performance across diverse food types. Despite these challenges, this study fills an important gap in food microbiology, offering a scalable solution for real-time food safety monitoring.

## Data Availability

The original contributions presented in the study are included in the article/supplementary materials, further inquiries can be directed to the corresponding authors.

## Supplementary Materials

The following supporting information can be downloaded at: https://www.mdpi.com/article/10.3390/foods15081277/s1, Table S1. Bacterial species used in this study; Table S2. LAMP primers targeting *HDC* gene of *Klebsiella aerogenes*, designed *de novo*; Table S3. GenBank accession numbers of *HDC* gene sequences from different *Klebsiella aerogenes* strains used for in silico alignment with PC2 primer; Table S4. *HDC*-producing *Enterobacteriaceae* strains and GenBank accession numbers of *HDC* gene sequences used for in silico specificity analysis of PC2 LAMP primers against *Klebsiella aerogenes HDC* target regions; Table S5. The results of biochemical tests for microbial identification of bacteria originating from food samples used in this study; Table S6. Results of the statistical analysis (one-way ANOVA followed by Tukey’s multiple comparisons test, *p* < 0.05) of the differences in Tt values of the *Klebsiella aerogenes* LAMP assay recorded for all tested food DNA samples obtained using the Chelex 100 approach; Table S7. Results of the statistical analysis (one-way ANOVA followed by Tukey’s multiple comparisons test, *p* < 0.05) of the differences in Tt values of the *Klebsiella aerogenes* LAMP assay recorded for vegetable DNA samples obtained using the Plant/Fungi DNA Isolation Kit; Table S8. Results of the statistical analysis (one-way ANOVA followed by Tukey’s multiple comparisons test, *p* < 0.05) of the differences in Tt values of the *Klebsiella aerogenes* LAMP assay recorded for meat DNA samples obtained using the DNeasy PowerFood Microbial Kit; Figure S1. Schematic illustration of *Klebsiella aerogenes HDC* gene showing LAMP primer positions and directions for primer candidate set 1 (PC1); Figure S2. Schematic illustration of *Klebsiella aerogenes HDC* gene showing LAMP primer positions and directions for primer candidate set 2 (PC2); Figure S3. Schematic illustration of *Klebsiella aerogenes HDC* gene showing LAMP primer positions and directions for primer candidate set 3 (PC3); Figure S4. *Klebsiella aerogenes* LAMP assay optimization (65 °C, 60 min); Figure S5. Real-time LAMP curves obtained using gDNA isolates derived from bacterial cultures cultivated from food samples for testing *Klebsiella aerogenes* contamination; Figure S6. Real-time LAMP curves showing the limit of detection of *Klebsiella aerogenes* in spiked vegetable and meat DNA extracts using the PC2 primer set after Chelex 100 DNA extraction; Figure S7. Real-time LAMP curves showing the limit of detection of *Klebsiella aerogenes* in spiked vegetable DNA extracts using the PC2 primer set after DNA extraction using the Plant/Fungi DNA Isolation Kit; Figure S8. Real-time LAMP curves showing the limit of detection of *Klebsiella aerogenes* in spiked meat DNA extracts using the PC2 primer set after DNA extraction using the DNeasy PowerFood Microbial Kit; Supplementary Data S1. Sequence alignment of *HDC* gene sequences from five *Klebsiella aerogenes* strains—Ka37751 (CP041925), NCTC9735 (LR134475), GY22PK002 (CP116606), C71872 (CP139379), isolate 57 (OW969633)—with PC2 LAMP primer sequences, confirming full complementarity within target regions; Supplementary Data S2. Sequence alignment of the LAMP assay target region of *Klebsiella aerogenes HDC* gene with *HDC* gene sequences from *HDC*-producing *Enterobacteriaceae*—*Klebsiella pneumoniae*, *Morganella morganii*, *Raoultella ornithinolytica, Raoultella planticola* and *Citrobacter youngae* (Table S4). Supplementary Data S3. Linear range analysis and variability of the LAMP assay for *Klebsiella aerogenes* gDNA. (a) Standard curve illustrating the relationship between time-to-threshold (Tt) and *K. aerogenes* gDNA concentration used to assess the linear detection range of the LAMP assay. (b) Table summarizing the coefficient of variation (CV) of Tt values calculated from replicate reactions during the LoD determination experiment.
