# Supplementary material for: A Rapid and Sensitive LAMP Assay for the Detection of Klebsiella aerogenes in Food Matrices
Source: Foods. 2026 Apr 8;15(8):1277. doi: 10.3390/foods15081277 (PMC13115305; doi:10.3390/foods15081277)
Supplement: Supplementary file 1 [file foods-15-01277-s001.zip › foods-4133749-supplementary.pdf]

Supplementary Information

A Rapid and Sensitive LAMP Assay for the Detection of *Klebsiella aerogenes* in Food Matrices

Mila Djisalov [1,*,†], Marija Pavlović [1,†], Ljiljana Janjušević [1], Ljiljana Šašić Zorić [1], Željko D. Popović [2] and Ivana Gadjanski [1,3,*]

[1] BioSense Institute, Center for Biosystems, University of Novi Sad, 21000 Novi Sad, Serbia
[2] Faculty of Sciences, University of Novi Sad, 21000 Novi Sad, Serbia; zeljko.popovic@dbe.uns.ac.rs
[3] Institute for Oncology and Radiology of Serbia, Department of Experimental Oncology, Belgrade 11000, Serbia
*Correspondence: mila.djisalov@biosense.rs (M.D.); ivana.gadjanski@ncrc.ac.rs (I.G.)
† These authors contributed equally to this work.

Summary

Table S1. Bacterial species used in this study.

Table S2. LAMP primers targeting *HDC* gene of *Klebsiella aerogenes*, designed *de novo.*

Table S3. GenBank accession numbers of *HDC* gene sequences from different *Klebsiella aerogenes* strains used for *in silico* alignment with PC2 primer.

Table S4. *HDC*-producing *Enterobacteriaceae* strains and GenBank accession numbers of *HDC* gene sequences used for *in silico* specificity analysis of PC2 LAMP primers against *Klebsiella aerogenes HDC* target regions.

Table S5. The results of biochemical tests for microbial identification of bacteria originating from food samples used in this study.

Table S6. Results of the statistical analysis (one-way ANOVA followed by Tukey's multiple comparisons test, $p < 0.05$) of the differences in Tt values of the *Klebsiella aerogenes* LAMP assay recorded for all tested food DNA samples obtained using the Chelex 100 approach.

Table S7. Results of the statistical analysis (one-way ANOVA followed by Tukey's multiple comparisons test, $p < 0.05$) of the differences in Tt values of the *Klebsiella aerogenes* LAMP assay recorded for vegetable DNA samples obtained using the Plant/Fungi DNA Isolation Kit.

Table S8. Results of the statistical analysis (one-way ANOVA followed by Tukey's multiple comparisons test, $p < 0.05$) of the differences in Tt values of the *Klebsiella aerogenes* LAMP assay recorded for meat DNA samples obtained using the DNeasy PowerFood Microbial Kit.

Figure S1. Schematic illustration of *Klebsiella aerogenes HDC* gene showing LAMP primer positions and directions for primer candidate set 1 (PC1).

Figure S2. Schematic illustration of *Klebsiella aerogenes HDC* gene showing LAMP primer positions and directions for primer candidate set 2 (PC2).

Figure S3. Schematic illustration of *Klebsiella aerogenes HDC* gene showing LAMP primer positions and directions for primer candidate set 3 (PC3).

Figure S4. *Klebsiella aerogenes* LAMP assay optimization (65°C, 60 min).

Figure S5. Real-time LAMP curves obtained using gDNA isolates derived from bacterial cultures cultivated from food samples for testing *Klebsiella aerogenes* contamination.

Figure S6. Real-time LAMP curves showing the limit of detection of *Klebsiella aerogenes* in spiked vegetable and meat DNA extracts using the PC2 primer set after Chelex 100 DNA extraction.

Figure S7. Real-time LAMP curves showing the limit of detection of *Klebsiella aerogenes* in spiked vegetable DNA extracts using the PC2 primer set after DNA extraction using the Plant/Fungi DNA Isolation Kit.

Figure S8. Real-time LAMP curves showing the limit of detection of *Klebsiella aerogenes* in spiked meat DNA extracts using the PC2 primer set after DNA extraction using the DNeasy PowerFood Microbial Kit.

Supplementary Data S1. Sequence alignment of *HDC* gene sequences from five *Klebsiella aerogenes* strains—Ka37751 (CP041925), NCTC9735 (LR134475), GY22PK002 (CP116606), C71872 (CP139379), isolate 57 (OW969633)—with PC2 LAMP primer sequences, confirming full complementarity within target regions.

Supplementary Data S2. Sequence alignment of the LAMP assay target region of *Klebsiella aerogenes HDC* gene with *HDC* gene sequences from *HDC*-producing *Enterobacteriaceae—Klebsiella pneumoniae, Morganella morganii*, *Raoultella ornithinolytica, Raoultella planticola* and *Citrobacter youngae* (Table S4).

Supplementary Data S3. Linear range analysis and variability of the LAMP assay for *Klebsiella aerogenes* gDNA. (a) Standard curve illustrating the relationship between time-to-threshold (Tt) and *K. aerogenes* gDNA concentration used to assess the linear detection range of the LAMP assay. (b) Table summarizing the coefficient of variation (CV) of Tt values calculated from replicate reactions during the LoD determination experiment.

Table S1. Bacterial species used in this study.

Bacterial species	Strain	ID number*
Klebsiella aerogenes	ATCC 13048	BSCCB-002
Escherichia coli	ATCC 25922	BSCCB-001
Salmonella enterica	13076	BSCCB-004
Staphylococcus aureus	ATCC 25923	BSCCB-003
Bacillus subtilis	PY79	BSCCB-007
Alcaligenes faecalis	In-house Bacterial Culture Collection of the BioSense Institute (BSCCB)	BSCCB-006

* The full table listing all bacterial species used in this study can be accessed via the BioSense Culture Collection (BSCC) at https://doi.org/10.5281/zenodo.18298613.

Table S2. LAMP primers targeting *HDC* gene of *Klebsiella aerogenes*, designed *de novo*.

Primer candidate (PC)	Primer name	Sequence 5'-3'	Position	Lengt h	GC rate	Tm
PC1-*HDC*	PC1-F3	ATTATCTACACGCGGATGCC	587-606	20	0.5	59.45
	PC1-B3	GCGTGACCCTGAAATCGT	799-816	18	0.56	59.18
	PC1-FIP (F1c + F2)	TGTCCGGAAACGCCAATCGAAT-TGATCTTGCCTTTCGTGGAG		42		
	PC1-BIP (B1c + B2)	TGGTAGCCAAGAAAGCCAACGT -TATCGTGGGCGGAGATGT		40		
	F2	TGATCTTGCCTTTCGTGGAG	620-639	20	0.5	59.51
	F1c	TGTCCGGAAACGCCAATCGAAT	674-695	22	0.5	64.83
	B2	TATCGTGGGCGGAGATGT	779-796	18	0.56	59.49
	B1c	TGGTAGCCAAGAAAGCCAACGT	731-752	22	0.5	64.57
	PC1-LF	GCGAAGGTAAACGGTTGTGGA	642-662	21	0.52	62.71
	PC1-LB	GACCGTATCAGCGTAGAGATCG	754-775	22	0.55	61.52

PC2- *HDC*	PC2-F3	ACGGTCATACCCCTTTGATG	818-837	20	0.5	59.04
	PC2-B3	CCAGGCAGTGTTTCTTCCAT	1005-1024	20	0.5	59.86
	PC2-FIP (F1c + F2)	CGGCGTATTTCGCCATGTTGAG-GTTCGCAGCCATACCGAT		40		
	PC2-BIP (B1c + B2)	AAGCAGCAGGTATTGACGCGC-CCCATTCAGAAGGCTTCGG		40		
	F2	GTTCGCAGCCATACCGAT	850-867	18	59.03	0.56
	F1c	CGGCGTATTTCGCCATGTTGAG	898-919	22	64.08	0.55
	B2	CCCATTCAGAAGGCTTCGG	985-1003	19	59.82	0.58
	B1c	AAGCAGCAGGTATTGACGCGC	932-952	21	65.68	0.57
	PC2-LF	GCTGTGACCAATGCGGC	881-897	17	60.79	0.65
	PC2-LB	CACAAAAACTCCATCACGGTGG	958-979	22	61.82	0.5
PC3- *HDC*	PC3-F3	CTCAACATGGCGAAATACGC	989-917	20	59.17	0.5
	PC3-B3	GCCAGGTCGGCAATTACG	1104-1121	18	60.26	0.61
	PC3-FIP (F1c + F2)	TCGGAAAGACCACCGTGATGGA -ATCGCTTTAAAGCAGCAGGT		42		
	PC3-BIP (B1c + B2)	CGTAGCCCATCTGATCACCACC-ATCGATCAGCGCATCAATCC		42		
	F2	ATCGCTTTAAAGCAGCAGGT	923-942	20	59.86	0.45
	F1c	TCGGAAAGACCACCGTGATGGA	967-988	22	65.52	0.55
	B2	ATCGATCAGCGCATCAATCC	1082-1101	20	60.06	0.5
	B1c	CGTAGCCCATCTGATCACCACC	1038-1059	22	64.45	0.59

	PC3-LB	TCACCACCTGGACAGTTCCC	1062-1081	20	63.37	0.6

Table S3. GenBank accession numbers of *HDC* gene sequences from different *Klebsiella aerogenes* strains used for *in silico* alignment with PC2 primer.

Klebsiella aerogenes	
**Strain**	**GenBank acc. No.**
Ka37751	CP041925
NCTC9735	LR134475
GY22PK002	CP116606
C71872	CP139379
isolate 57	OW969633

Table S4. *HDC*-producing *Enterobacteriaceae* strains and GenBank accession numbers of *HDC* gene sequences used for in silico specificity analysis of PC2 LAMP primers against *Klebsiella aerogenes HDC* target regions.

***HDC*-producing bacteria**	**Strain**	**Accession No.**
Klebsiella aerogenes	Ka37751	CP041925
Klebsiella pneumoniae	GDFK0932	CP167296
Klebsiella pneumoniae	PB270	NZ_FLVO01000025
Morganella morganii	DI-244	KP728802
Morganella morganii	ATCC 9237	KP728801
Raoultella ornithinolytica	RoM27LC23	CP130153
Raoultella ornithinolytica	HPP19	FJ469566
Raoultella ornithinolytica	HPP15	FJ469565
Raoultella planticola	RP_3045	CP114772
Raoultella planticola	S8	AB075221
Raoultella planticola	Y1-1	AB075220
Citrobacter youngae	NCTC8782	UIGT01000001
Citrobacter youngae	BB1468	CAHPRB010000005

Table S5. The results of biochemical tests for microbial identification of bacteria originating from food samples used in this study.

SAMPLE NO.	SAMPLE SOURCE	GRAM STAINING	MORPHOLOGY	NITRATE REDUCTASE TEST	CATALASE TEST	OXIDASE TEST	INDOLE TEST	VOGES-PROSCAUER TEST	METYL-RED TEST
1.	***E. coli* (control)**	-	rod-shaped	+	+	-	+	-	+
2.	***K. aerogenes* (control)**	-	rod-shaped	+	+	-	-	+	-
3.	**Lettuce-1**	+	cocci	-	+	+	-	-	-
4.	**Lettuce-2 (*Bacillus*)**	+	Not tested	Not tested	Not tested	Not tested	Not tested	Not tested	Not tested
5.	**Carrot-1**	+	rod-shaped	+	+	+	-	-	+
6.	**Carrot-2**	-	rod-shaped	+	+	+	-	-	+
7.	**Cucumber-1 (*Bacillus*)**	+	Not tested	Not tested	Not tested	Not tested	Not tested	Not tested	Not tested
8.	**Cucumber-2 (*Bacillus*)**	+	Not tested	Not tested	Not tested	Not tested	Not tested	Not tested	Not tested
9.	**Chicken breasts-1**	+	cocci	+	+	-	-	-	+
10.	**Chicken breasts-2**	+	cocci	-	+	-	-	-	+
11.	**Chicken breasts-3 (*Bacillus*)**	+	Not tested	Not tested	Not tested	Not tested	Not tested	Not tested	Not tested
12	**Salami-1**	+	cocci	+	+	-	-	-	+
13.	**Salami-2**	+	cocci	+	+	-	-	-	+

Table S6. Results of the statistical analysis (one-way ANOVA followed by Tukey's multiple comparisons test, $p < 0.05$) of the differences in Tt values of the *Klebsiella aerogenes* LAMP assay recorded for all tested food DNA samples obtained using the Chelex 100 approach.

	**Samples**	**Mean Diff.**	**95.00% CI of diff.**	**Significant?**	**Summary**	**P Value**
**food DNA extracts spiked with 1 ng/μL of *K. aerogenes* gDNA**	Carrot 1 vs. Cucumber 1	1.383	-1.342 to 4.109	No	ns	0.4567
	Carrot 1 vs. Lettuce 1	1.2	-1.525 to 3.925	No	ns	0.5779
	Carrot 1 vs. Chicken breasts 1	0.7917	-2.255 to 3.839	No	ns	0.8901
	Carrot 1 vs. Salami 1	1.292	-1.755 to 4.339	No	ns	0.6088
	Cucumber 1 vs. Lettuce 1	-0.1833	-2.909 to 2.542	No	ns	0.9992
	Cucumber 1 vs. Chicken breasts 1	-0.5917	-3.639 to 2.455	No	ns	0.9575
	Cucumber 1 vs. Salami 1	-0.09167	-3.139 to 2.955	No	ns	>0.9999
	Lettuce 1 vs. Chicken breasts 1	-0.4083	-3.455 to 2.639	No	ns	0.9888
	Lettuce 1 vs. Salami 1	0.09167	-2.955 to 3.139	No	ns	>0.9999
	Chicken breasts 1 vs. Salami 1	0.5	-2.838 to 3.838	No	ns	0.9831
**food DNA extracts spiked with 1 x 10^{-1} ng/μL of *K. aerogenes* gDNA**	Carrot 1 x 10^{-1} vs. Cucumber 1 x 10^{-1}	1.617	-1.177 to 4.411	No	ns	0.3454
	Carrot 1 x 10^{-1} vs. Lettuce 1 x 10^{-1}	1.9	-0.8941 to 4.694	No	ns	0.223
	Carrot 1 x 10^{-1} vs. Chicken breasts 1 x 10^{-1}	0.4667	-2.657 to 3.591	No	ns	0.9832
	Carrot 1 x 10^{-1} vs. Salami 1 x 10^{-1}	3.042	-0.08229 to 6.166	No	ns	0.0566

	Samples	Mean Diff.	95.00% CI of diff.	Significant?	Summary	P Value
	Cucumber 1 x 10^{-1} vs. Lettuce 1 x 10^{-1}	0.2833	-2.511 to 3.077	No	ns	0.9961
	Cucumber 1 x 10^{-1} vs. Chicken breasts 1 x 10^{-1}	-1.15	-4.274 to 1.974	No	ns	0.7141
	Cucumber 1 x 10^{-1} vs. Salami 1 x 10^{-1}	1.425	-1.699 to 4.549	No	ns	0.5484
	Lettuce 1 x 10^{-1} vs. Chicken breasts 1 x 10^{-1}	-1.433	-4.557 to 1.691	No	ns	0.5434
	Lettuce 1 x 10^{-1} vs. Salami 1 x 10^{-1}	1.142	-1.982 to 4.266	No	ns	0.719
	Chicken breasts 1 x 10^{-1} vs. Salami 1 x 10^{-1}	2.575	-0.8471 to 5.997	No	ns	0.1603
**food DNA extracts spiked with 1 x 10^{-2} ng/µL of *K. aerogenes* gDNA**	Carrot 1 x 10^{-2} vs. Cucumber 1 x 10^{-2}	2.733	-2.126 to 7.593	No	ns	0.3689
	Carrot 1 x 10^{-2} vs. Lettuce 1 x 10^{-2}	4.317	-0.5425 to 9.176	No	ns	0.0847
	Carrot 1 x 10^{-2} vs. Chicken breasts 1 x 10^{-2}	-1.317	-6.749 to 4.116	No	ns	0.9115
	Carrot 1 x 10^{-2} vs. Salami 1 x 10^{-2}	5.408	-0.02439 to 10.84	No	ns	0.0511
	Cucumber 1 x 10^{-2} vs. Lettuce 1 x 10^{-2}	1.583	-3.276 to 6.443	No	ns	0.7898
	Cucumber 1 x 10^{-2} vs. Chicken breasts 1 x 10^{-2}	-4.05	-9.483 to 1.383	No	ns	0.1655
	Cucumber 1 x 10^{-2} vs. Salami 1 x 10^{-2}	2.675	-2.758 to 8.108	No	ns	0.483

	Samples	Mean Diff.	95.00% CI of diff.	Significant?	Summary	P Value
	Lettuce 1 x 10^{-2} vs. Chicken breasts 1 x 10^{-2}	-5.633	-11.07 to -0.2006	Yes	*	0.0421
	Lettuce 1 x 10^{-2} vs. Salami 1 x 10^{-2}	1.092	-4.341 to 6.524	No	ns	0.9522
	Chicken breasts 1 x 10^{-2} vs. Salami 1 x 10^{-2}	6.725	0.7738 to 12.68	Yes	*	0.0273

Table S7. Results of the statistical analysis (one-way ANOVA followed by Tukey's multiple comparisons test, $p < 0.05$) of the differences in Tt values of the *Klebsiella aerogenes* LAMP assay recorded for vegetable DNA samples obtained using the Plant/Fungi DNA Isolation kit.

	Samples	Mean Diff.	95.00% CI of diff.	Significant?	Summary	P Value
**vegetable DNA extracts spiked with 1 ng/µL of *K. aerogenes* gDNA**	Carrot 1 vs. Lettuce 1	0.075	-7.672 to 7.822	No	ns	0.9991

Table S8. Results of the statistical analysis (one-way ANOVA followed by Tukey's multiple comparisons test, $p < 0.05$) of the differences in Tt values of the *Klebsiella aerogenes* LAMP assay recorded for meat DNA samples obtained using the DNeasy PowerFood Microbial Kit.

	**Samples**	**Mean Diff.**	**95.00% CI of diff.**	**Significant?**	**Summary**	**P Value**
**meat DNA extracts spiked with 1 ng/μL of *K. aerogenes* gDNA**	Chicken breasts 1 vs. Salami 1	0.8	-2.017 to 3.617	No	ns	0.6502
**meat DNA extracts spiked with 1 x 10^{-1} ng/μL of *K. aerogenes* gDNA**	Chicken breasts 1 x 10^{-1} vs. Salami 1 x 10^{-1}	-1.867	-5.066 to 1.333	No	ns	0.2027
**meat DNA extracts spiked with 1 x 10^{-2} ng/μL of *K. aerogenes* gDNA**	Chicken breasts 1 x 10^{-2} vs. Salami 1 x 10^{-2}	-0.3	-4.072 to 3.472	No	ns	0.9572

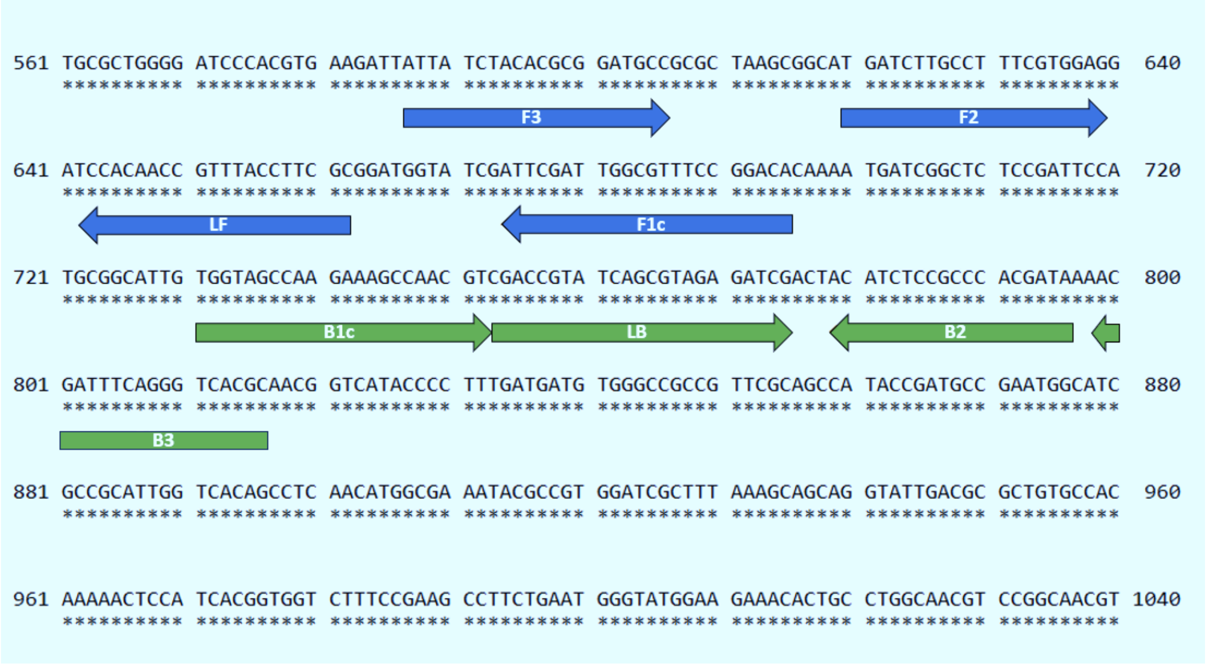

Figure S1. Schematic illustration of *Klebsiella aerogenes HDC* gene showing LAMP primer positions and directions for primer candidate set 1 (PC1).

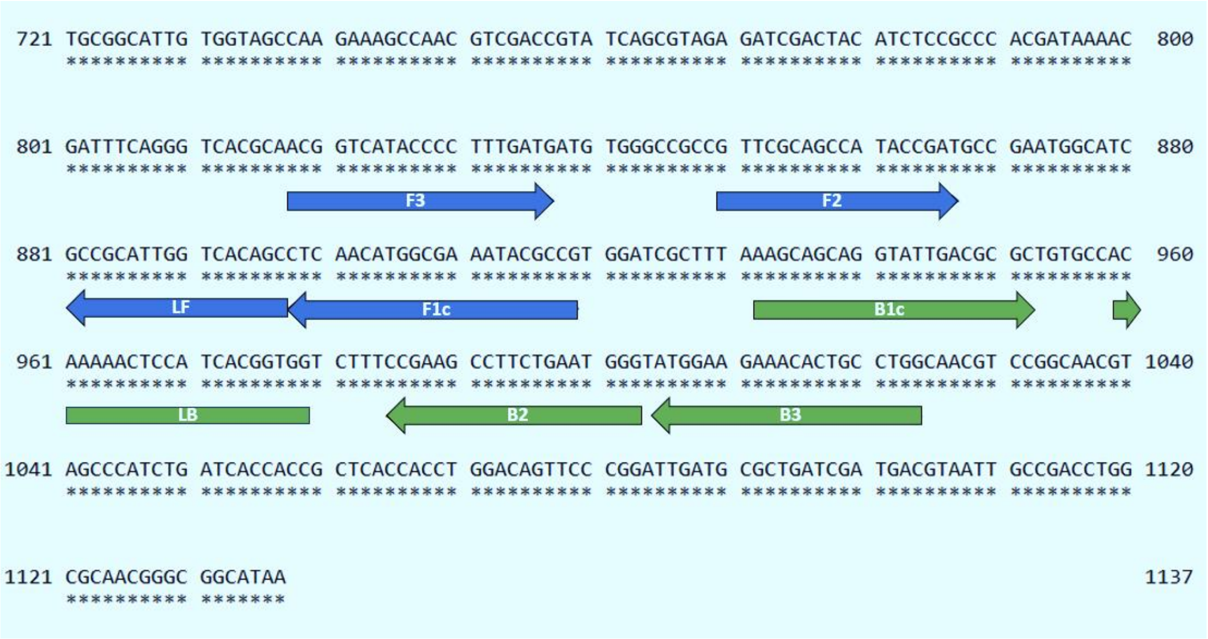

Figure S2. Schematic illustration of *Klebsiella aerogenes HDC* gene showing LAMP primer positions and directions for primer candidate set 2 (PC2).

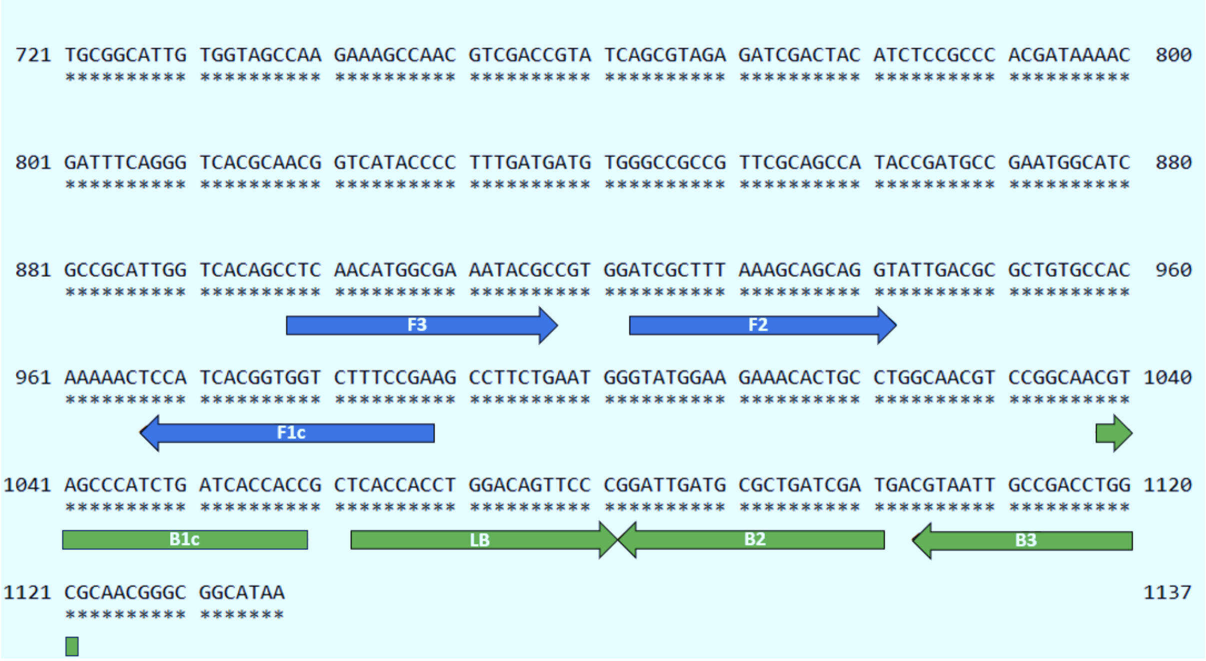

Figure S3. Schematic illustration of *Klebsiella aerogenes HDC* gene showing LAMP primer positions and directions for primer candidate set 3 (PC3).

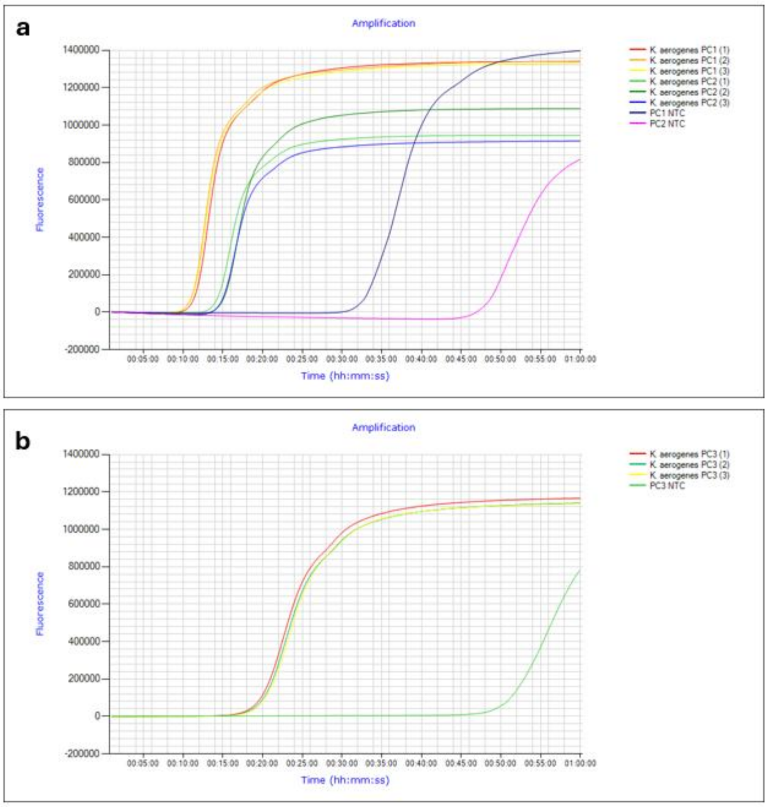

Figure S4. *Klebsiella aerogenes* LAMP assay optimization (65°C, 60 min): (a) PC1-PC2; (b) PC3 primer sets (*K. aerogenes* gDNA vs. NTC).

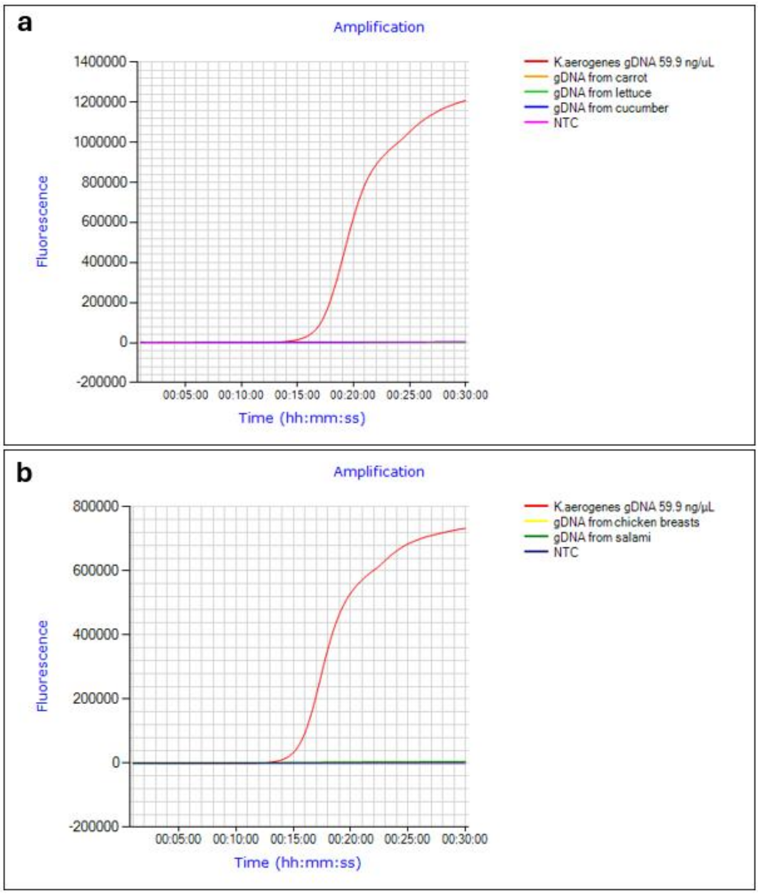

Figure S5. Real-time LAMP curves obtained using gDNA isolates derived from bacterial cultures cultivated from food samples for testing *Klebsiella aerogenes* contamination. (a) Vegetable samples and (b) meat samples. Red curves represent the positive control (*K. aerogenes* gDNA).

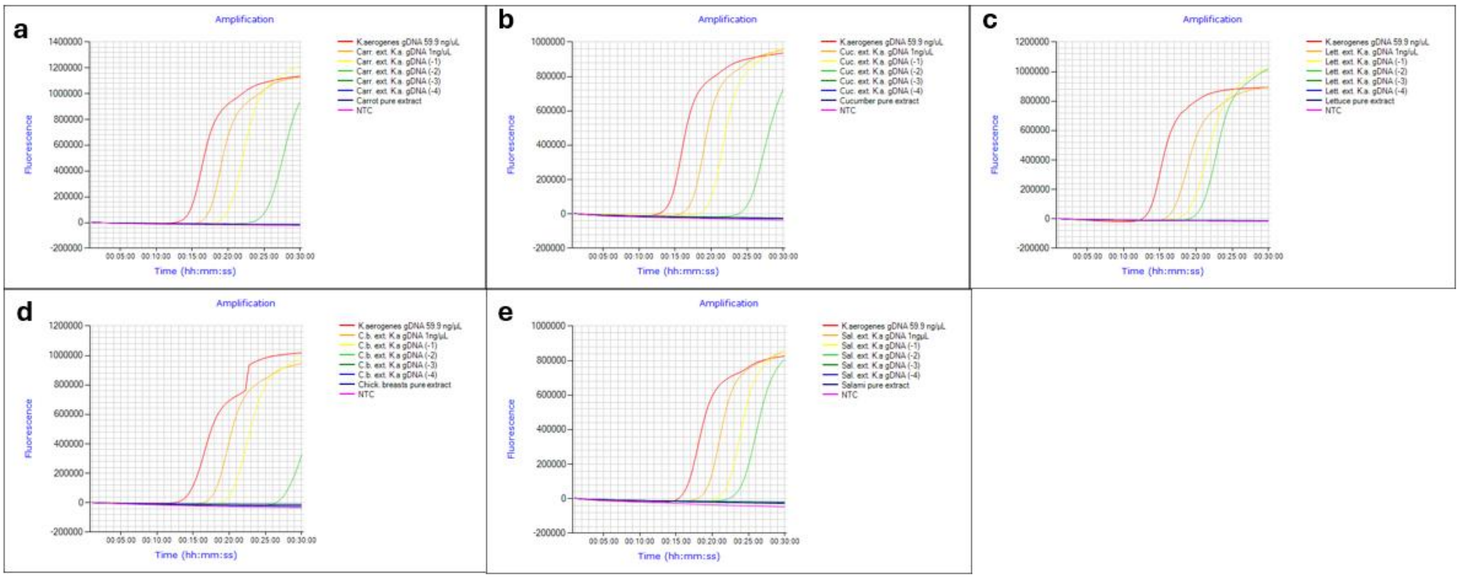

Figure S6. Real-time LAMP curves showing the limit of detection of *Klebsiella aerogenes* in spiked vegetable and meat DNA extracts using the PC2 primer set after Chelex 100 DNA extraction. a) Carrot DNA extracts spiked with *K. aerogenes* gDNA at concentrations of 1, 0.1, 0.01, 0.001, and 0.0001 ng/µL: red curve, positive control (59.9 ng/µL *K. aerogenes* gDNA); orange, 1 ng/µL; yellow, 0.1 ng/µL; light green, 0.01 ng/µL; dark green, 0.001 ng/µL; light blue, 0.0001 ng/µL; dark blue, non-spiked carrot DNA; pink, no-template control (NTC). b) Cucumber DNA extracts spiked with the same concentrations of *K. aerogenes* gDNA as in spiked carrot DNA extracts: color code same as in a). c) Lettuce DNA extracts spiked with the same concentrations of *K. aerogenes* gDNA as in spiked carrot DNA extracts; color code same as in a). d) Chicken breast DNA extracts spiked with the same concentrations of *K. aerogenes* gDNA as in spiked carrot DNA extracts; color code same as in a). e) Salami DNA extracts spiked with the same concentrations of *K. aerogenes* gDNA as in spiked carrot DNA extracts; color code same as in a).

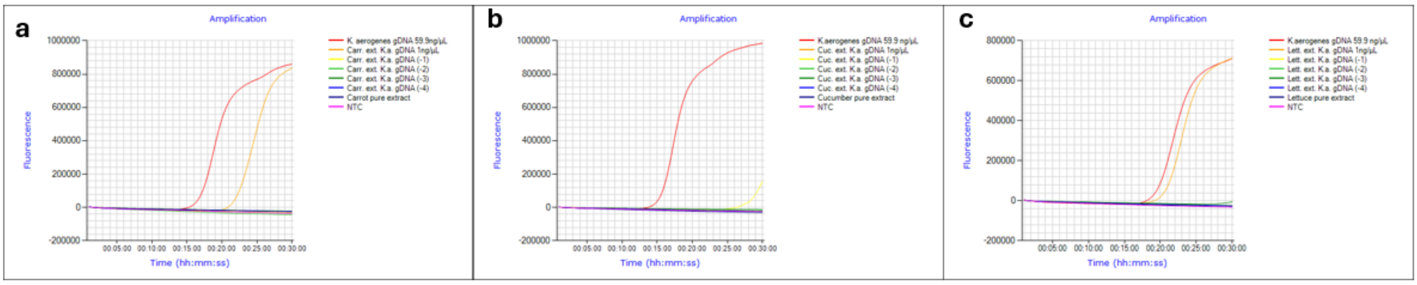

Figure S7. Real-time LAMP curves showing the limit of detection of *Klebsiella aerogenes* in spiked vegetable DNA extracts using the PC2 primer set after DNA extraction using the Plant/Fungi DNA Isolation Kit. a) Carrot DNA extracts spiked with *K. aerogenes* gDNA at concentrations of 1, 0.1, 0.01, 0.001, and 0.0001 ng/µL: red curve, positive control (59.9 ng/µL *K. aerogenes* gDNA); orange, 1 ng/µL; yellow, 0.1 ng/µL; light green, 0.01 ng/µL; dark green, 0.001 ng/µL; light blue, 0.0001 ng/µL; dark blue, non-spiked carrot DNA; pink, no-template control (NTC). b) Cucumber DNA extracts spiked with the same concentrations of *K. aerogenes* gDNA as in spiked carrot DNA extracts; color code same as in a). c) Lettuce DNA extracts spiked with the same concentrations of *K. aerogenes* as in spiked carrot DNA extracts; color code same as in a).

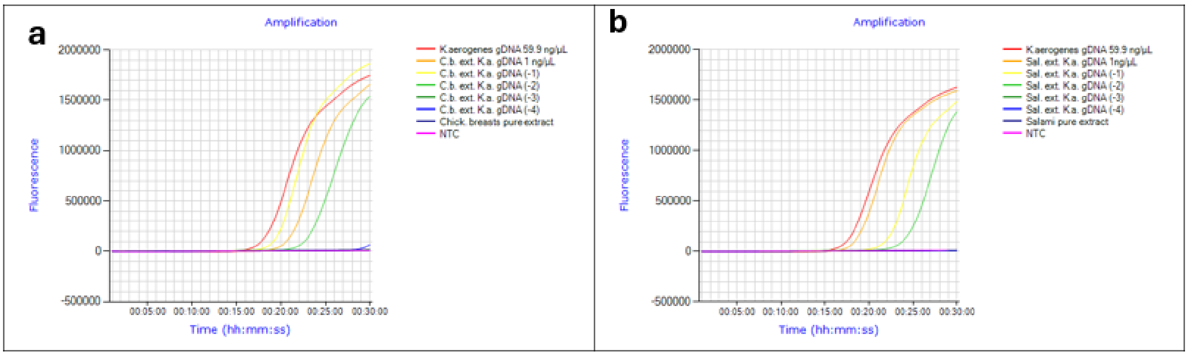

Figure S8. Real-time LAMP curves showing the limit of detection of *Klebsiella aerogenes* in spiked meat DNA extracts using the PC2 primer set after DNA extraction using the DNeasy PowerFood Microbial Kit. a) Chicken breast DNA extracts spiked with *K. aerogenes* gDNA at concentrations of 1, 0.1, 0.01, 0.001, and 0.0001 ng/µL: red curve, positive control (59.9 ng/µL *K. aerogenes* gDNA); orange, 1 ng/µL; yellow, 0.1 ng/µL; light green, 0.01 ng/µL; dark green, 0.001 ng/µL; light blue, 0.0001 ng/µL; dark blue, non-spiked chicken breast DNA; pink, no-template control (NTC). b) Salami DNA extracts spiked with the same concentrations of *K. aerogenes* gDNA as in spiked chicken breast DNA extracts; color code same as in a).

```
                      10         20         30         40         50         60         70         80         90        100
              ....|....| ....|....| ....|....| ....|....| ....|....| ....|....| ....|....| ....|....| ....|....| ....|....|
Ka37751       ATGTCTTTAT CTATTGCCGA TCAAAATAAA CTCGATGCAT TTTGGTCATA CTGTGTAAAA AATCGCTACT TTAATATTGG CTACCCTGAA TCTGCTGATT
NCTC9735      ATGTCTTTAT CTATTGCCGA TCAAAATAAA CTCGATGCAT TTTGGTCATA CTGTGTAAAA AATCGCTACT TTAATATTGG CTACCCTGAA TCCGCTGATT
GY22PK002     ATGTCTTTAT CTATTGCCGA TCAAAATAAA CTCGATGCAT TTTGGTCATA CTGTGTAAAA AATCGCTACT TTAATATTGG CTACCCTGAA TCTGCTGATT
C71872        ATGTCTTTAT CTATTGCCGA TCAAAATAAA CTCGATGCAT TTTGGTCATA CTGTGTAAAA AATCGCTACT TTAATATTGG CTACCCTGAA TCTGCTGATT
isolate 57    ATGTCTTTAT CTATTGCCGA TCAAAATAAA CTCGATGCAT TTTGGTCATA CTGTGTAAAA AATCGCTACT TTAATATTGG CTACCCTGAA TCTGCTGATT
PC2 F3        ---------- ---------- ---------- ---------- ---------- ---------- ---------- ---------- ---------- ----------
PC2 F1c (r.)  ---------- ---------- ---------- ---------- ---------- ---------- ---------- ---------- ---------- ----------
PC2 LF (r.)   ---------- ---------- ---------- ---------- ---------- ---------- ---------- ---------- ---------- ----------
PC2 F2        ---------- ---------- ---------- ---------- ---------- ---------- ---------- ---------- ---------- ----------
PC2 B3 (r.)   ---------- ---------- ---------- ---------- ---------- ---------- ---------- ---------- ---------- ----------
PC2 B1c       ---------- ---------- ---------- ---------- ---------- ---------- ---------- ---------- ---------- ----------
PC2 B2 (r.)   ---------- ---------- ---------- ---------- ---------- ---------- ---------- ---------- ---------- ----------
PC2 LB        ---------- ---------- ---------- ---------- ---------- ---------- ---------- ---------- ---------- ----------

                     110        120        130        140        150        160        170        180        190        200
              ....|....| ....|....| ....|....| ....|....| ....|....| ....|....| ....|....| ....|....| ....|....| ....|....|
Ka37751       TTGATTACAC CATGCTGGAG CGTTTTCTGC GTTTCTCAAT TAATAACTGT GGTGACTGGG GGGAGTACTG TAACTATTTA CTCAACTCTT TCGACTTTGA
NCTC9735      TTGATTACAC CATGCTGGAG CGTTTTCTGC GTTTCTCAAT TAATAACTGT GGTGACTGGG GGGAGTACTG TAACTATTTA CTCAACTCTT TCGACTTTGA
GY22PK002     TTGATTACAC CATGCTGGAG CGTTTTCTGC GTTTCTCAAT TAATAACTGT GGTGACTGGG GGGAGTACTG TAACTATTTA CTCAACTCTT TCGACTTTGA
C71872        TTGATTACAC CATGCTGGAG CGTTTTCTGC GTTTCTCAAT TAATAACTGT GGTGACTGGG GGGAGTACTG TAACTATTTA CTCAACTCTT TCGACTTTGA
isolate 57    TTGATTACAC CATGCTGGAG CGTTTTCTGC GTTTCTCAAT TAATAACTGT GGTGACTGGG GGGAGTACTG TAACTATTTA CTCAACTCTT TCGACTTTGA
PC2 F3        ---------- ---------- ---------- ---------- ---------- ---------- ---------- ---------- ---------- ----------
PC2 F1c (r.)  ---------- ---------- ---------- ---------- ---------- ---------- ---------- ---------- ---------- ----------
PC2 LF (r.)   ---------- ---------- ---------- ---------- ---------- ---------- ---------- ---------- ---------- ----------
PC2 F2        ---------- ---------- ---------- ---------- ---------- ---------- ---------- ---------- ---------- ----------
PC2 B3 (r.)   ---------- ---------- ---------- ---------- ---------- ---------- ---------- ---------- ---------- ----------
PC2 B1c       ---------- ---------- ---------- ---------- ---------- ---------- ---------- ---------- ---------- ----------
PC2 B2 (r.)   ---------- ---------- ---------- ---------- ---------- ---------- ---------- ---------- ---------- ----------
PC2 LB        ---------- ---------- ---------- ---------- ---------- ---------- ---------- ---------- ---------- ----------

                     210        220        230        240        250        260        270        280        290        300
              ....|....| ....|....| ....|....| ....|....| ....|....| ....|....| ....|....| ....|....| ....|....| ....|....|
Ka37751       AAAAGAAGTC ATGGAGTATT TCTCCGGCAT ATTCAAAATC CCCTTTGCGG AAAGCTGGGG CTATGTCACC AACGGCGGCA CAGAAAGTAA TATGTTTGGT
NCTC9735      AAAAGAAGTC ATGGAGTATT TCTCCGGCAT ATTCAAAATC TCCTTTGCGG AAAGCTGGGG CTATGTCACC AACGGCGGCA CAGAAAGTAA TATGTTTGGT
GY22PK002     AAAAGAAGTC ATGGAGTATT TCTCCGGCAT ATTCAAAATC CCCTTTGCGG AAAGCTGGGG CTATGTCACC AACGGCGGCA CAGAAAGTAA TATGTTTGGT
C71872        AAAAGAAGTC ATGGAGTATT TCTCCGGCAT ATTCAAAATC CCCTTTGCGG AAAGCTGGGG CTATGTCACC AACGGCGGCA CAGAAAGTAA TATGTTTGGT
isolate 57    AAAAGAAGTC ATGGAGTATT TCTCCGGCAT ATTCAAAATC CCCTTTGCGG AAAGCTGGGG CTATGTCACC AACGGCGGCA CAGAAAGTAA TATGTTTGGT
PC2 F3        ---------- ---------- ---------- ---------- ---------- ---------- ---------- ---------- ---------- ----------
PC2 F1c (r.)  ---------- ---------- ---------- ---------- ---------- ---------- ---------- ---------- ---------- ----------
PC2 LF (r.)   ---------- ---------- ---------- ---------- ---------- ---------- ---------- ---------- ---------- ----------
PC2 F2        ---------- ---------- ---------- ---------- ---------- ---------- ---------- ---------- ---------- ----------
PC2 B3 (r.)   ---------- ---------- ---------- ---------- ---------- ---------- ---------- ---------- ---------- ----------
PC2 B1c       ---------- ---------- ---------- ---------- ---------- ---------- ---------- ---------- ---------- ----------
PC2 B2 (r.)   ---------- ---------- ---------- ---------- ---------- ---------- ---------- ---------- ---------- ----------
PC2 LB        ---------- ---------- ---------- ---------- ---------- ---------- ---------- ---------- ---------- ----------

                     310        320        330        340        350        360        370        380        390        400
              ....|....| ....|....| ....|....| ....|....| ....|....| ....|....| ....|....| ....|....| ....|....| ....|....|
Ka37751       TGCTATCTGG GCAGAGAGCT GTTCCCGGAA GGCACACTCT ACTATTCAAA AGATACTCAC TATTCCGTCG CCAAAATCGT CAAACTGCTG CGTATCAAAT
NCTC9735      TGCTATCTGG GCAGAGAGCT GTTCCCGGAA GGCACACTCT ACTATTCAAA AGATACTCAC TATTCCGTCG CCAAAATCGT CAAACTGCTG CGTATCAAAT
GY22PK002     TGCTATCTGG GCAGAGAGCT GTTCCCGGAA GGCACGCTCT ACTATTCAAA AGATACTCAC TATTCCGTCG CCAAAATCGT CAAACTGCTG CGTATCAAAT
C71872        TGCTATCTGG GAAGAGAACT GTTCCCGGAA GGCACGCTCT ACTATTCAAA AGATACTCAC TATTCCGTCG CCAAAATCGT CAAACTGCTG CGTATCAAAT
isolate 57    TGCTATCTGG GAAGAGAACT GTTCCCGGAA GGCACGCTCT ACTATTCAAA AGATACTCAC TATTCCGTCG CCAAAATCGT CAAACTGCTG CGTATCAAAT
PC2 F3        ---------- ---------- ---------- ---------- ---------- ---------- ---------- ---------- ---------- ----------
PC2 F1c (r.)  ---------- ---------- ---------- ---------- ---------- ---------- ---------- ---------- ---------- ----------
PC2 LF (r.)   ---------- ---------- ---------- ---------- ---------- ---------- ---------- ---------- ---------- ----------
PC2 F2        ---------- ---------- ---------- ---------- ---------- ---------- ---------- ---------- ---------- ----------
PC2 B3 (r.)   ---------- ---------- ---------- ---------- ---------- ---------- ---------- ---------- ---------- ----------
PC2 B1c       ---------- ---------- ---------- ---------- ---------- ---------- ---------- ---------- ---------- ----------
PC2 B2 (r.)   ---------- ---------- ---------- ---------- ---------- ---------- ---------- ---------- ---------- ----------
PC2 LB        ---------- ---------- ---------- ---------- ---------- ---------- ---------- ---------- ---------- ----------

                     410        420        430        440        450        460        470        480        490        500
              ....|....| ....|....| ....|....| ....|....| ....|....| ....|....| ....|....| ....|....| ....|....| ....|....|
Ka37751       CGCAACTGGT GGAATCTCAG CCAGACGGAG AAATGGATTA TGACGATTTG ATCAATAAAA TCAGGACCTC AGGCGAACGC CATCCCATCA TTTTCGCCAA
NCTC9735      CGCAACTGGT GGAATCTCAG CCAGACGGAG AAATGGATTA TGACGATTTG ATCAATAAAA TCAGGACCTC AGGCGAACGC CATCCCATCA TTTTCGCCAA
GY22PK002     CGCAACTGGT GGAGTCTCAG CCAGACGGAG AAATGGATTA TGACGATTTG ATCAATAAAA TCAGGACCTC AGGCGAACGC CATCCCATCA TTTTCGCCAA
C71872        CGCAACTGGT GGAATCTCAG CCAGACGGAG AAATGGATTA TGACGATTTG ATCAATAAAA TCAGGACCTC AGGCGAACGC CATCCCATCA TTTTCGCCAA
isolate 57    CGCAACTGGT GGAATCTCAG CCAGACGGAG AAATGGATTA TGACGATTTG ATCAATAAAA TCAGGACCTC AGGCGAACGC CATCCCATCA TTTTCGCCAA
PC2 F3        ---------- ---------- ---------- ---------- ---------- ---------- ---------- ---------- ---------- ----------
PC2 F1c (r.)  ---------- ---------- ---------- ---------- ---------- ---------- ---------- ---------- ---------- ----------
PC2 LF (r.)   ---------- ---------- ---------- ---------- ---------- ---------- ---------- ---------- ---------- ----------
PC2 F2        ---------- ---------- ---------- ---------- ---------- ---------- ---------- ---------- ---------- ----------
PC2 B3 (r.)   ---------- ---------- ---------- ---------- ---------- ---------- ---------- ---------- ---------- ----------
PC2 B1c       ---------- ---------- ---------- ---------- ---------- ---------- ---------- ---------- ---------- ----------
PC2 B2 (r.)   ---------- ---------- ---------- ---------- ---------- ---------- ---------- ---------- ---------- ----------
PC2 LB        ---------- ---------- ---------- ---------- ---------- ---------- ---------- ---------- ---------- ----------

                     510        520        530        540        550        560        570        580        590        600
              ....|....| ....|....| ....|....| ....|....| ....|....| ....|....| ....|....| ....|....| ....|....| ....|....|
Ka37751       TATTGGCACA ACGGTACGCG GCGCTGTTGA TAATATCGCT GAGATACAGA AACGTATCGC TGCGCTGGGG ATCCCACGTG AAGATTATTA TCTACACGCG
NCTC9735      TATTGGCACA ACGGTACGCG GCGCTGTTGA TAATATCGCT GAGATACAGA AACGTATCGC TGCGCTGGGG ATCCCACGTG AAGATTATTA TCTACACGCG
```

```
GY22PK002      TATTGGCACA ACGGTACGCG GCGCTGTTGA TAATATCGCT GAGATACAGA AACGTATCGC TGCGCTGGGG ATCCCACGTG AAGATTATTA TCTACACGCG
C71872         TATTGGCACA ACGGTACGCG GCGCTGTTGA TAATATCGCT GAGATACAGA AACGTATCGC TGCGCTGGGG ATCCCACGTG AAGATTATTA TCTACACGCG
isolate 57     TATTGGCACA ACGGTACGCG GCGCTGTTGA TAATATCGCT GAGATACAGA AACGTATCGC TGCGCTGGGG ATCCCACGTG AAGATTATTA TCTACACGCG
PC2 F3         ---------- ---------- ---------- ---------- ---------- ---------- ---------- ---------- ---------- ----------
PC2 F1c (r.)   ---------- ---------- ---------- ---------- ---------- ---------- ---------- ---------- ---------- ----------
PC2 LF (r.)    ---------- ---------- ---------- ---------- ---------- ---------- ---------- ---------- ---------- ----------
PC2 F2         ---------- ---------- ---------- ---------- ---------- ---------- ---------- ---------- ---------- ----------
PC2 B3 (r.)    ---------- ---------- ---------- ---------- ---------- ---------- ---------- ---------- ---------- ----------
PC2 B1c        ---------- ---------- ---------- ---------- ---------- ---------- ---------- ---------- ---------- ----------
PC2 B2 (r.)    ---------- ---------- ---------- ---------- ---------- ---------- ---------- ---------- ---------- ----------
PC2 LB         ---------- ---------- ---------- ---------- ---------- ---------- ---------- ---------- ---------- ----------

                      610        620        630        640        650        660        670        680        690        700
               ....|....| ....|....| ....|....| ....|....| ....|....| ....|....| ....|....| ....|....| ....|....| ....|....|
Ka37751        GATGCCGCGC TAAGCGGCAT GATCTTGCCT TTCGTGGAGG ATCCACAACC GTTTACCTTC GCGGATGGTA TCGATTCGAT TGGCGTTTCC GGACACAAAA
NCTC9735       GATGCCGCGC TAAGCGGCAT GATCTTGCCT TTCGTGGAGG ATCCACAACC GTTTACCTTC GCGGATGGTA TCGATTCGAT TGGCGTTTCC GGACACAAAA
GY22PK002      GATGCCGCGC TAAGCGGCAT GATCTTGCCT TTCGTGGAGG ATCCACAACC GTTTACCTTC GCGGATGGTA TCGATTCGAT TGGCGTTTCC GGACACAAAA
C71872         GATGCCGCGC TAAGCGGCAT GATCTTGCCT TTCGTGGAGG ATCCACAACC GTTTACCTTC GCGGATGGTA TCGATTCGAT TGGCGTTTCC GGACACAAAA
isolate 57     GATGCCGCGC TAAGCGGCAT GATCTTGCCT TTCGTGGAGG ATCCACAACC GTTTACCTTC GCGGATGGTA TCGATTCGAT TGGCGTTTCC GGACACAAAA
PC2 F3         ---------- ---------- ---------- ---------- ---------- ---------- ---------- ---------- ---------- ----------
PC2 F1c (r.)   ---------- ---------- ---------- ---------- ---------- ---------- ---------- ---------- ---------- ----------
PC2 LF (r.)    ---------- ---------- ---------- ---------- ---------- ---------- ---------- ---------- ---------- ----------
PC2 F2         ---------- ---------- ---------- ---------- ---------- ---------- ---------- ---------- ---------- ----------
PC2 B3 (r.)    ---------- ---------- ---------- ---------- ---------- ---------- ---------- ---------- ---------- ----------
PC2 B1c        ---------- ---------- ---------- ---------- ---------- ---------- ---------- ---------- ---------- ----------
PC2 B2 (r.)    ---------- ---------- ---------- ---------- ---------- ---------- ---------- ---------- ---------- ----------
PC2 LB         ---------- ---------- ---------- ---------- ---------- ---------- ---------- ---------- ---------- ----------

                      710        720        730        740        750        760        770        780        790        800
               ....|....| ....|....| ....|....| ....|....| ....|....| ....|....| ....|....| ....|....| ....|....| ....|....|
Ka37751        TGATCGGCTC TCCGATTCCA TGCGGCATTG TGGTAGCCAA GAAAGCCAAC GTCGACCGTA TCAGCGTAGA GATCGACTAC ATCTCCGCCC ACGATAAAAC
NCTC9735       TGATCGGCTC TCCGATTCCA TGCGGCATTG TGGTAGCCAA GAAAGCCAAC GTCGACCGTA TCAGCGTAGA GATCGACTAC ATCTCCGCCC ACGATAAAAC
GY22PK002      TGATCGGCTC TCCGATTCCA TGCGGCATTG TGGTAGCCAA GAAAGCCAAC GTCGACCGTA TCAGCGTAGA GATCGACTAC ATCTCCGCCC ACGATAAAAC
C71872         TGATCGGCTC TCCGATTCCA TGCGGCATTG TGGTAGCCAA GAAAGCCAAC GTCGACCGTA TCAGCGTAGA GATCGACTAC ATCTCCGCCC ACGATAAAAC
isolate 57     TGATCGGCTC TCCGATTCCA TGCGGCATTG TGGTAGCCAA GAAAGCCAAC GTCGACCGTA TCAGCGTAGA GATCGACTAC ATCTCCGCCC ACGATAAAAC
PC2 F3         ---------- ---------- ---------- ---------- ---------- ---------- ---------- ---------- ---------- ----------
PC2 F1c (r.)   ---------- ---------- ---------- ---------- ---------- ---------- ---------- ---------- ---------- ----------
PC2 LF (r.)    ---------- ---------- ---------- ---------- ---------- ---------- ---------- ---------- ---------- ----------
PC2 F2         ---------- ---------- ---------- ---------- ---------- ---------- ---------- ---------- ---------- ----------
PC2 B3 (r.)    ---------- ---------- ---------- ---------- ---------- ---------- ---------- ---------- ---------- ----------
PC2 B1c        ---------- ---------- ---------- ---------- ---------- ---------- ---------- ---------- ---------- ----------
PC2 B2 (r.)    ---------- ---------- ---------- ---------- ---------- ---------- ---------- ---------- ---------- ----------
PC2 LB         ---------- ---------- ---------- ---------- ---------- ---------- ---------- ---------- ---------- ----------

                      810        820        830        840        850        860        870        880        890        900
               ....|....| ....|....| ....|....| ....|....| ....|....| ....|....| ....|....| ....|....| ....|....| ....|....|
Ka37751        GATTTCAGGG TCACGCAACG GTCATACCCC TTTGATGATG TGGGCCGCCG TTCGCAGCCA TACCGATGCC GAATGGCATC GCCGCATTGG TCACAGCCTC
NCTC9735       GATTTCAGGG TCACGCAACG GTCATACCCC TTTGATGATG TGGGCCGCCG TTCGCAGCCA TACCGATGCC GAATGGCATC GCCGCATTGG TCACAGCCTC
GY22PK002      GATTTCAGGG TCACGCAACG GTCATACCCC TTTGATGATG TGGGCCGCCG TTCGCAGCCA TACCGATGCC GAATGGCATC GCCGCATTGG TCACAGCCTC
C71872         GATTTCAGGG TCACGCAACG GTCATACCCC TTTGATGATG TGGGCCGCCG TTCGCAGCCA TACCGATGCC GAATGGCATC GCCGCATTGG TCACAGCCTC
isolate 57     GATTTCAGGG TCACGCAACG GTCATACCCC TTTGATGATG TGGGCCGCCG TTCGCAGCCA TACCGATGCC GAATGGCATC GCCGCATTGG TCACAGCCTC
PC2 F3         ---------- -------ACG GTCATACCCC TTTGATG--- ---------- ---------- ---------- ---------- ---------- ----------
PC2 F1c (r.)   ---------- ---------- ---------- ---------- ---------- ---------- ---------- ---------- ---------- -------CTC
PC2 LF (r.)    ---------- ---------- ---------- ---------- ---------- ---------- ---------- ---------- GCCGCATTGG TCACAGC---
PC2 F2         ---------- ---------- ---------- ---------- ---------G TTCGCAGCCA TACCGAT--- ---------- ---------- ----------
PC2 B3 (r.)    ---------- ---------- ---------- ---------- ---------- ---------- ---------- ---------- ---------- ----------
PC2 B1c        ---------- ---------- ---------- ---------- ---------- ---------- ---------- ---------- ---------- ----------
PC2 B2 (r.)    ---------- ---------- ---------- ---------- ---------- ---------- ---------- ---------- ---------- ----------
PC2 LB         ---------- ---------- ---------- ---------- ---------- ---------- ---------- ---------- ---------- ----------

                      910        920        930        940        950        960        970        980        990       1000
               ....|....| ....|....| ....|....| ....|....| ....|....| ....|....| ....|....| ....|....| ....|....| ....|....|
Ka37751        AACATGGCGA AATACGCCGT GGATCGCTTT AAAGCAGCAG GTATTGACGC GCTGTGCCAC AAAAACTCCA TCACGGTGGT CTTTCCGAAG CCTTCTGAAT
NCTC9735       AACATGGCGA AATACGCCGT GGATCGCTTT AAAGCAGCAG GTATTGACGC GCTGTGCCAC AAAAACTCCA TCACGGTGGT CTTTCCGAAG CCTTCTGAAT
GY22PK002      AACATGGCGA AATACGCCGT GGATCGCTTT AAAGCAGCAG GTATTGACGC GCTGTGCCAC AAAAACTCCA TCACGGTGGT CTTTCCGAAG CCTTCTGAAT
C71872         AACATGGCGA AATACGCCGT GGATCGCTTT AAAGCAGCAG GTATTGACGC GCTGTGCCAC AAAAACTCCA TCACGGTGGT CTTTCCGAAG CCTTCTGAAT
isolate 57     AACATGGCGA AATACGCCGT GGATCGCTTT AAAGCAGCAG GTATTGACGC GCTGTGCCAC AAAAACTCCA TCACGGTGGT CTTTCCGAAG CCTTCTGAAT
PC2 F3         ---------- ---------- ---------- ---------- ---------- ---------- ---------- ---------- ---------- ----------
PC2 F1c (r.)   AACATGGCGA AATACGCCG- ---------- ---------- ---------- ---------- ---------- ---------- ---------- ----------
PC2 LF (r.)    ---------- ---------- ---------- ---------- ---------- ---------- ---------- ---------- ---------- ----------
PC2 F2         ---------- ---------- ---------- ---------- ---------- ---------- ---------- ---------- ---------- ----------
PC2 B3 (r.)    ---------- ---------- ---------- ---------- ---------- ---------- ---------- ---------- ---------- ----------
PC2 B1c        ---------- ---------- ---------- -AAGCAGCAG GTATTGACGC GC-------- ---------- ---------- ---------- ----------
PC2 B2 (r.)    ---------- ---------- ---------- ---------- ---------- ---------- ---------- ---------- ----CCGAAG CCTTCTGAAT
PC2 LB         ---------- ---------- ---------- ---------- ---------- -------CAC AAAAACTCCA TCACGGTGG- ---------- ----------

                     1010       1020       1030       1040       1050       1060       1070       1080       1090       1100
               ....|....| ....|....| ....|....| ....|....| ....|....| ....|....| ....|....| ....|....| ....|....| ....|....|
Ka37751        GGGTATGGAA GAAACACTGC CTGGCAACGT CCGGCAACGT AGCCCATCTG ATCACCACCG CTCACCACCT GGACAGTTCC CGGATTGATG CGCTGATCGA
NCTC9735       GGGTATGGAA GAAACACTGC CTGGCAACGT CCGGCAACGT AGCCCATCTG ATCACCACCG CTCACCACCT GGACAGTTCC CGGATTGATG CGCTGATCGA
GY22PK002      GGGTATGGAA GAAACACTGC CTGGCAACGT CCGGCAACGT GGCCCATCTG ATCACCACCG CTCACCACCT GGACAGTTCC CGGATTGATG CGCTGATCGA
C71872         GGGTATGGAA GAAACACTGC CTGGCAACGT CCGGCAACGT AGCCCATCTG ATCACCACCG CTCACCACCT GGACAGTTCC CGGATTGATG CGCTGATCGA
isolate 57     GGGTATGGAA GAAACACTGC CTGGCAACGT CCGGCAACGT AGCCCATCTG ATCACCACCG CTCACCACCT GGACAGTTCC CGGATTGATG CGCTGATCGA
PC2 F3         ---------- ---------- ---------- ---------- ---------- ---------- ---------- ---------- ---------- ----------

PC2 F1c (r.)   ---------- ---------- ---------- ---------- ---------- ---------- ---------- ---------- ---------- ----------
PC2 LF (r.)    ---------- ---------- ---------- ---------- ---------- ---------- ---------- ---------- ---------- ----------
PC2 F2         ---------- ---------- ---------- ---------- ---------- ---------- ---------- ---------- ---------- ----------
PC2 B3 (r.)    ----ATGGAA GAAACACTGC CTGG------ ---------- ---------- ---------- ---------- ---------- ---------- ----------
PC2 B1c        ---------- ---------- ---------- ---------- ---------- ---------- ---------- ---------- ---------- ----------
PC2 B2 (r.)    GGG------- ---------- ---------- ---------- ---------- ---------- ---------- ---------- ---------- ----------
PC2 LB         ---------- ---------- ---------- ---------- ---------- ---------- ---------- ---------- ---------- ----------
```

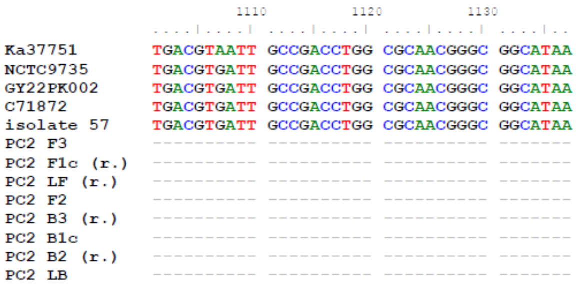

Supplementary Data S1. Sequence alignment of *HDC* gene sequences from five *Klebsiella aerogenes* strains—Ka37751 (CP041925), NCTC9735 (LR134475), GY22PK002 (CP116606), C71872 (CP139379), isolate 57 (OW969633)—with PC2 LAMP primer sequences, confirming full complementarity within target regions. Sequence alignment was performed using BioEdit v7.2.0 software (Informer Technologies, Inc.).

```
                                          10         20         30         40         50         60         70         80
                                  ....|....| ....|....| ....|....| ....|....| ....|....| ....|....| ....|....| ....|....|
K. aerogenes st. Ka37751          ATGTCTTTAT CTATTGCCGA TCAAAATAAA CTCGATGCAT TTTGGTCATA CTGTGTAAAA AATCGCTACT TTAATATTGG
K. pneumoniae st. GDFK0932        ATGTCTTTAT CATTTGAAGA TCAACACAAA CTTGATGAGT TCTGGTCTTA TTGTGTCAAA CATCAGTATT TCAATATTGG
K. pneumoniae st. PB270           ATGTCTTTAT CATTTGAAGA TCAACACAAA CTTGATGAGT TCTGGTCTTA TTGTGTCAAA CATCAGTATT TCAATATTGG
M. morganii st. DI-244            ATGACTCTGT CTATCAATGA TCAAAACAAA CTTGATGCAT TCTGGGCTTA TTGCGTAAAA AACCAGTATT TCAACATCGG
M. morganii st. ATCC 9237         ATGACTCTGT CTATCAATGA TCAAAACAAA CTTGATGCAT TCTGGGCTTA TTGCGTAAAA AACCAGTATT TCAACATCGG
R. ornithinolytica st. RoM27LC23  ATGACATTAT CCATTAGCGA TCAAAATAAA CTTGATTCTT TCTGGTCATA TTGTGTAAAA AACCAATATT TTAATATCGG
R. ornithinolytica st. HPP19      ---------- ---------- ---------- ---------- ---------- ---------- ---------- ----------
R. ornithinolytica st. HPP15      ---------- ---------- ---------- ---------- ---------- ---------- ---------- ----------
Raoultella planticola st. RP_3045 ATGACATTAT CCATTAGCGA TCAAAATAAA CTTGATTCTT TCTGGTCATA TTGTGTAAAA AACCAATATT TTAATATCGG
R. planticola st. S8              ---------- ---------- ---------- ---------- ---------- ---------- ---------- ----------
R. planticola st. Y1-1            ---------- ---------- ---------- ---------- ---------- ---------- ---------- ----------
C. youngae st. NCTC8782           ATGACATTAT CTATTGTCGA TCAGAATAAG CTCGATGCTT TTTGGTCATA TTGCGTAAAA AATCAGTATT TTAATATAGG
C. youngae isolate BB1468         ATGACATTAT CTATTGTCGA TCAGAATAAG CTCGATGCTT TTTGGTCATA TTGCGTAAAA AATCAGTATT TTAATATAGG
PC2 F3                            ---------- ---------- ---------- ---------- ---------- ---------- ---------- ----------
PC2 F1c (r.)                      ---------- ---------- ---------- ---------- ---------- ---------- ---------- ----------
PC2 LF (r.)                       ---------- ---------- ---------- ---------- ---------- ---------- ---------- ----------
PC2 F2                            ---------- ---------- ---------- ---------- ---------- ---------- ---------- ----------
PC2 B3 (r.)                       ---------- ---------- ---------- ---------- ---------- ---------- ---------- ----------
PC2 B1c                           ---------- ---------- ---------- ---------- ---------- ---------- ---------- ----------
PC2 B2 (r.)                       ---------- ---------- ---------- ---------- ---------- ---------- ---------- ----------
PC2 LB                            ---------- ---------- ---------- ---------- ---------- ---------- ---------- ----------

                                          90        100        110        120        130        140        150        160
                                  ....|....| ....|....| ....|....| ....|....| ....|....| ....|....| ....|....| ....|....|
K. aerogenes st. Ka37751          CTACCCTGAA TCTGCTGATT TTGATTACAC CATGCTGGAG CGTTTTCTGC GTTTCTCAAT TAATAACTGT GGTGACTGGG
K. pneumoniae st. GDFK0932        TTACCCAGAA TCTGCTGATT TCAATTACAC AGTTCCGGAA CGTTTCATGC GTTTTTCCAT CAACAACTGT GGTGACTGGG
K. pneumoniae st. PB270           TTACCCAGAA TCTGCTGATT TCAATTACAC AGTTCCGGAA CGTTTCATGC GTTTTTCCAT CAACAACTGT GGTGACTGGG
M. morganii st. DI-244            CTATCCTGAA TCAGCAGATT TCGATTACAC CAACCTGGAA CGTTTCTTAC GTTTCTCCAT CAACAACTGC GGTGACTGGG
M. morganii st. ATCC 9237         CTATCCTGAA TCAGCGGATT TCGATTACAC CAACCTGGAA CGTTTTTTAC GTTTCTCCAT CAACAACTGC GGTGACTGGG
R. ornithinolytica st. RoM27LC23  ATATCCTGAA TCGGCAGATT TTGATTACAC CATCCTTGAA CGTTTCATGC GCTTCTCAAT CAACAACTGT GGTGACTGGG
R. ornithinolytica st. HPP19      ---------- ---------- ---------- ---------- ---------- -----TCTAT CAGCAACTGT GGTGACTGGG
R. ornithinolytica st. HPP15      ---------- ---------- ---------- ---------- ---------- -----TCCAT CAGTAACTGT GGTGACTGGG
Raoultella planticola st. RP_3045 ATATCCTGAA TCGGCAGATT TTGATTACAC CATCCTTGAA CGTTTCATGC GCTTCTCAAT CAACAACTGT GGCGATTGGG
R. planticola st. S8              ---------- ---------- ---------- ---------- ---------- ---------- ---------- ----------
R. planticola st. Y1-1            ---------- ---------- ---------- ---------- ---------- ---------- ---------- ----------
C. youngae st. NCTC8782           TTATCCGGAG TCAGCCGACT TCGATTACAC GATACTGGAA CGGTTTATGC GTTTCTCCAT AAATAATTGT GGTGACTGGG
C. youngae isolate BB1468         TTATCCGGAG TCAGCCGACT TCGATTACAC GATACTGGAA CGGTTTATGC GTTTCTCCAT AAATAATTGT GGTGACTGGG
PC2 F3                            ---------- ---------- ---------- ---------- ---------- ---------- ---------- ----------
PC2 F1c (r.)                      ---------- ---------- ---------- ---------- ---------- ---------- ---------- ----------
PC2 LF (r.)                       ---------- ---------- ---------- ---------- ---------- ---------- ---------- ----------
PC2 F2                            ---------- ---------- ---------- ---------- ---------- ---------- ---------- ----------
PC2 B3 (r.)                       ---------- ---------- ---------- ---------- ---------- ---------- ---------- ----------
PC2 B1c                           ---------- ---------- ---------- ---------- ---------- ---------- ---------- ----------
PC2 B2 (r.)                       ---------- ---------- ---------- ---------- ---------- ---------- ---------- ----------
PC2 LB                            ---------- ---------- ---------- ---------- ---------- ---------- ---------- ----------

                                         170        180        190        200        210        220        230        240
                                  ....|....| ....|....| ....|....| ....|....| ....|....| ....|....| ....|....| ....|....|
K. aerogenes st. Ka37751          GGGAGTACTG TAACTATTTA CTCAACTCTT TCGACTTTGA AAAAGAAGTC ATGGAGTATT TCTCCGGCAT ATTCAAAATC
K. pneumoniae st. GDFK0932        CTGATTACTG TAATTACCGG CTGAATACCT TTGATTTTGA AAAAGAGGTT ATAGCCTACT TTGCTGGGGT GTTCAAAATT
K. pneumoniae st. PB270           CTGATTACTG TAATTACCGG CTGAATACCT TTGATTTTGA AAAAGAGGTT ATAGCCTACT TTGCTGGGGT GTTCAAAATT
M. morganii st. DI-244            GCGAATATTG CAACTACCTG CTGAACTCTT TCGATTTCGA GAAAGAAGTG ATGGAGTATT TCGCAGACCT GTTCAAAATT
M. morganii st. ATCC 9237         GCGAATATTG CAACTACCTG CTGAACTCTT TCGATTTCGA GAAAGAAGTG ATGGAGTATT TCGCAGACCT GTTCAAAATT
R. ornithinolytica st. RoM27LC23  GGGAATACTG CAACTACTTA TTAAACTCCT TTGATTTTGA AAAAGAGGTC ATGGAGTATT TCGCCCAGCT ATTTAAAATC
R. ornithinolytica st. HPP19      GGGAATACTG CAACTACTTA TTAAACTCCT TTGATTTTGA AAAAGAGGTC ATGGAGTATT TCGCCCAGCT ATTTAAAATC
R. ornithinolytica st. HPP15      GGGAATACTG CAACTACTTA TTAAACTCCT TTGATTTTGA AAAAGAGGTC ATGGAGTATT TCGCCCAGCT ATTTAAAATC
Raoultella planticola st. RP_3045 GAGAATACTG CAACTACTTA TTAAACTCCT TTGATTTTGA AAAAGAGGTC ATGGAGTATT TCGCCCAGCT ATTTAAAATC
R. planticola st. S8              ---------- ---------- ---------- ---------- ---------- ---------- ---------- ----------
R. planticola st. Y1-1            ---------- ---------- ---------- ---------- ---------- ---------- ---------- ----------
C. youngae st. NCTC8782           GGGAGTATTG CAATTATTTG CTGAACTCGT TTGATTTTGA AAAAGAAGTA ATGGAGTATT TCGCCAGTAT TTTCAAAATC
C. youngae isolate BB1468         GGGAGTATTG CAATTATTTG CTGAACTCGT TTGATTTTGA AAAAGAAGTA ATGGAGTATT TCGCCAGTAT TTTCAAAATC
PC2 F3                            ---------- ---------- ---------- ---------- ---------- ---------- ---------- ----------
PC2 F1c (r.)                      ---------- ---------- ---------- ---------- ---------- ---------- ---------- ----------
PC2 LF (r.)                       ---------- ---------- ---------- ---------- ---------- ---------- ---------- ----------
PC2 F2                            ---------- ---------- ---------- ---------- ---------- ---------- ---------- ----------
PC2 B3 (r.)                       ---------- ---------- ---------- ---------- ---------- ---------- ---------- ----------
PC2 B1c                           ---------- ---------- ---------- ---------- ---------- ---------- ---------- ----------
PC2 B2 (r.)                       ---------- ---------- ---------- ---------- ---------- ---------- ---------- ----------
PC2 LB                            ---------- ---------- ---------- ---------- ---------- ---------- ---------- ----------

                                         250        260        270        280        290        300        310        320
                                  ....|....| ....|....| ....|....| ....|....| ....|....| ....|....| ....|....| ....|....|
K. aerogenes st. Ka37751          CCCTTTGCGG AAAGCTGGGG CTATGTCACC AACGGCGGCA CAGAAAGTAA TATGTTTGGT TGCTATCTGG GCAGAGAGCT
K. pneumoniae st. GDFK0932        CCATTTGAGC AGTGTTGGGG CTATGTAACA AATGGGGGGA CAGAAGGTAA CATGTTCGGA TGTTATCTGG GACGTGAATT
K. pneumoniae st. PB270           CCATTTGAGC AGTGTTGGGG CTATGTAACA AATGGGGGGA CAGAAGGTAA CATGTTCGGA TGTTATCTGG GACGTGAATT
M. morganii st. DI-244            CCGTTTGAAC AAAGCTGGGG TTATGTGACC AACGGCGGTA CTGAAGGTAA CATGTTCGGT TGCTACCTGG GCCGTGAAAT
M. morganii st. ATCC 9237         CCGTTTGAAA AAAGCTGGGG TTATGTGACC AACGGCGGTA CTGAAGGTAA CATGTTCGGT TGCTACCTGG GCCGTGAAAT
R. ornithinolytica st. RoM27LC23  CCATTTGAAG AAAGCTGGGG GTATGTGACC AATGGCGGTA CCGAAGGCAA TATGTTTGGC TGCTATCTGG GTCGGGAGAT
R. ornithinolytica st. HPP19      CCATTTGAAG AAAGCTGGGG GTATGTGACC AATGGCGGTA CCGAAGGCAA TATGTTTGGC TGCTATCTGG GTCGGGAGAT
R. ornithinolytica st. HPP15      CCATTTGAAG AAAGCTGGGG GTATGTGACC AATGGCGGTA CCGAAGGCAA TATGTTTGGC TGCTATCTGG GTCGGGAGAT
Raoultella planticola st. RP_3045 CCATTTGAAG AAAGTTGGGG GTATGTGACC AATGGCGGTA CCGAAGGCAA TATGTTTGGC TGCTATCTGG GTCGGGAGAT
R. planticola st. S8              ---------- ---------- ---------- AATGGCGGTA CCGAAGGCAA TATGTTTGGC TGCTATCTGG GTCGGGAGAT
```

```
R. planticola st. Y1-1               ---------- ---------- ---------- AATGGCGGTA CCGAAGGCAA TATGTTTGGC TGCTATCTGG GTCGGGAGAT
C. youngae st. NCTC8782              CCATTTGAAG AAAGCTGGGG TTACGTTACC AATGGGGGAA CTGAAGGCAA TATGTTTGGC TGCTATCTGG GGCGTGAACT
C. youngae isolate BB1468            CCATTTGAAG AAAGCTGGGG TTACGTTACC AATGGGGGAA CTGAAGGTAA TATGTTTGGC TGCTATCTGG GGCGTGAACT
PC2 F3                               ---------- ---------- ---------- ---------- ---------- ---------- ---------- ----------
PC2 F1c (r.)                         ---------- ---------- ---------- ---------- ---------- ---------- ---------- ----------
PC2 LF (r.)                          ---------- ---------- ---------- ---------- ---------- ---------- ---------- ----------
PC2 F2                               ---------- ---------- ---------- ---------- ---------- ---------- ---------- ----------
PC2 B3 (r.)                          ---------- ---------- ---------- ---------- ---------- ---------- ---------- ----------
PC2 B1c                              ---------- ---------- ---------- ---------- ---------- ---------- ---------- ----------
PC2 B2 (r.)                          ---------- ---------- ---------- ---------- ---------- ---------- ---------- ----------
PC2 LB                               ---------- ---------- ---------- ---------- ---------- ---------- ---------- ----------

                                            330        340        350        360        370        380        390        400
                                     ....|....| ....|....| ....|....| ....|....| ....|....| ....|....| ....|....| ....|....|
K. aerogenes st. Ka37751             GTTCCCGGAA GGCACACTCT ACTATTCAAA AGATACTCAC TATTCCGTCG CCAAAATCGT CAAACTGCTG CGTATCAAAT
K. pneumoniae st. GDFK0932           ATTTCCTGAT GCCATACTTT ACTACTCCAG AGACACTCAC TACTCAGTTG CAAAAATAGT CAAATTGCTC CGAATCAAAT
K. pneumoniae st. PB270              ATTTCCTGAT GCCATACTTT ACTACTCCAG AGACACTCAC TACTCAGTTG CAAAAATAGT CAAATTGCTC CGAATCAAAT
M. morganii st. DI-244               CTTCCCTGAC GGTACCCTGT ACTATTCAAA AGATACTCAC TATTCCGTTG CGAAAATCGT TAAATTACTG CGTATCAAAT
M. morganii st. ATCC 9237            CTTCCCTGAC GGTACCCTGT ACTATTCAAA AGATACTCAC TATTCCGTTG CGAAAATCGT TAAATTACTG CGTATCAAAT
R. ornithinolytica st. RoM27LC23     TTTCCCCAAC GGTACACTAT ACTACTCGAA AGATACCCAT TACTCCGTGG CAAAAATCGT CAAGCTGTTG CGCATAAAAT
R. ornithinolytica st. HPP19         TTTCCCCAAC GGTACACTAT ACTACTCGAA AGATACCCAT TACTCCGTGG CAAAAATCGT CAAGCTGTTG CGCATAAAAT
R. ornithinolytica st. HPP15         TTTCCCCAAC GGTACACTAT ACTACTCGAA AGATACCCAT TACTCCGTGG CAAAAATCGT CAAGCTGTTG CGCATAAAAT
Raoultella planticola st. RP_3045    TTTCCCCAAC GGTACGCTAT ACTACTCGAA AGATACCCAT TACTCCGTGG CAAAAATCGT CAAACTGTTG CGCATAAAAT
R. planticola st. S8                 TTTCCCCAAC GGTACACTAT ACTACTCGAA AGATACCCAT TACTCCGTGG CAAAAATCGT CAAGCTGTTG CGCATAAAAT
R. planticola st. Y1-1               TTTCCCCAAC GGTACACTAT ACTACTCGAA AGATACCCAT TACTCCGTGG CAAAAATCGT CAAGCTGTTA CGCATAAAAT
C. youngae st. NCTC8782              GTTTCCAGAA GGTACGCTTT ATTACTCGAA AGATACTCAT TATTCCGTTG CGAAAATTGT TAAGTTGTTG CGCATTAAAT
C. youngae isolate BB1468            GTTTCCAGAA GGTACGCTTT ATTACTCGAA AGATACTCAT TATTCCGTTG CGAAAATTGT TAAGTTGTTG CGCATTAAAT
PC2 F3                               ---------- ---------- ---------- ---------- ---------- ---------- ---------- ----------
PC2 F1c (r.)                         ---------- ---------- ---------- ---------- ---------- ---------- ---------- ----------
PC2 LF (r.)                          ---------- ---------- ---------- ---------- ---------- ---------- ---------- ----------
PC2 F2                               ---------- ---------- ---------- ---------- ---------- ---------- ---------- ----------
PC2 B3 (r.)                          ---------- ---------- ---------- ---------- ---------- ---------- ---------- ----------
PC2 B1c                              ---------- ---------- ---------- ---------- ---------- ---------- ---------- ----------
PC2 B2 (r.)                          ---------- ---------- ---------- ---------- ---------- ---------- ---------- ----------
PC2 LB                               ---------- ---------- ---------- ---------- ---------- ---------- ---------- ----------

                                            410        420        430        440        450        460        470        480
                                     ....|....| ....|....| ....|..|.| ....|....| ....|....| ....|....| ....|....| ....|....|
K. aerogenes st. Ka37751             CGCAACTGGT GGAATCTCAG CCAGACGGAG AAATGGATTA TGACGATTTG ATCAATAAAA TCAGGACCTC AGGCGAACGC
K. pneumoniae st. GDFK0932           CACAGGTAGT CGAATCTCAG CCAAACGGTG AAATGGATTA CGATGATCTG ATGAAAAAAA TATCCGTTGA TAGAGAAACT
K. pneumoniae st. PB270              CACAGGTAGT CGAATCTCAG CCAAACGGTG AAATGGATTA CGATGATCTG ATGAAAAAAA TATCCGTTGA TAGAGAAACT
M. morganii st. DI-244               CTCAGGTTGT TGAATCTCTG CCAAACGGCG AAATCGACTA TGACGATCTG ATGAAAAAAA TCGCTGACGA TAAAGAAGCG
M. morganii st. ATCC 9237            CTCAGGTTGT TGAATCTCAG CCAAACGGCG AAATCGACTA TGACGATCTG ATGAAAAAAA TCGCTGACGA TAAAGAAGCG
R. ornithinolytica st. RoM27LC23     CGACGCTTGT TGAATCGCAG CCCAATGGTG AGATGGATTA TGCCGATTTA ATCAAAAAAA TAAAACGCGA CAATGAAAAG
R. ornithinolytica st. HPP19         CGACGCTTGT TGAATCGCAG CCCAATGGTG AGATGGATTA TGCCGATTTA ATCAAAAAAA TAAAACGCGA CAATGAAAAG
R. ornithinolytica st. HPP15         CGACGCTTGT TGAATCGCAG CCCAATGGTG AGATGGATTA TGCCGATTTA ATCAAAAAAA TAAAACGCGA CAATGAAAAG
Raoultella planticola st. RP_3045    CGACACTTGT TGAATCGCAA CCCAATGGTG AGATGGATTA CACCGATTTA ATCAAAAAAA TAAAACGCGA CAATGAAAAA
R. planticola st. S8                 CGACGCTTGT TGAATCGCAG CCCAATGGTG AGATGGATTA TGCCGATTTA ATCAAAAAAA TAAAACGCGA CAATGAAAAG
R. planticola st. Y1-1               CGACGCTTGT TGAATCGCAG CCCAATGGTG AGATGGATTA TGCCGATTTA ATCAAAAAAA TAAAACGCGA CAATGAAAAG
C. youngae st. NCTC8782              CAAGTCTGGT GGAATCACAG CCGAATGGTG AGATGGACTA TGATGATTTG ATTCGTAAAA TTCAACGCGA TAACGAAGAA
C. youngae isolate BB1468            CAAGTCTGGT GGAATCACAG CCGAATGGTG AGATGGACTA TGATGATTTG ATTCGTAAAA TTCAACGCGA TAACGAAGAA
PC2 F3                               ---------- ---------- ---------- ---------- ---------- ---------- ---------- ----------
PC2 F1c (r.)                         ---------- ---------- ---------- ---------- ---------- ---------- ---------- ----------
PC2 LF (r.)                          ---------- ---------- ---------- ---------- ---------- ---------- ---------- ----------
PC2 F2                               ---------- ---------- ---------- ---------- ---------- ---------- ---------- ----------
PC2 B3 (r.)                          ---------- ---------- ---------- ---------- ---------- ---------- ---------- ----------
PC2 B1c                              ---------- ---------- ---------- ---------- ---------- ---------- ---------- ----------
PC2 B2 (r.)                          ---------- ---------- ---------- ---------- ---------- ---------- ---------- ----------
PC2 LB                               ---------- ---------- ---------- ---------- ---------- ---------- ---------- ----------

                                            490        500        510        520        530        540        550        560
                                     ....|....| ....|....| ....|....| ....|....| ....|....| ....|....| ....|....| ....|....|
K. aerogenes st. Ka37751             CATCCCATCA TTTTCGCCAA TATTGGCACA ACGGTACGCG GCGCTGTTGA TAATATCGCT GAGATACAGA AACGTATCGC
K. pneumoniae st. GDFK0932           AATCCAATAA TATTTGCTAA TATTGGTTCT ACAGTACGTG GGGCGATAGA TAATATTGAC GAAATTCAAA AACGTTTGCA
K. pneumoniae st. PB270              AATCCAATAA TATTTGCTAA TATTGGTTCT ACAGTACGTG GGGCGATAGA TAATATTGAC GAAATTCAAA AACGTTTGCA
M. morganii st. DI-244               CATCCGATCA TCTTCGCTAA CATCGGTACC ACTGTCCGCG GTGCTATTGA TGATATCGCT GAAATCCAGA AACGCCTGAA
M. morganii st. ATCC 9237            CATCCGATCA TCTTCGCTAA CATCGGTACC ACTGTCCGCG GTGCTATTGA TGATATCGCT GAAATCCAGA AACGCCTGAA
R. ornithinolytica st. RoM27LC23     CACCCCATTA TTTTTGCAAA TATCGGGACT ACCGTTCGCG GAGCTATTGA TAATATTGCC ATCATTCAGC AATCAATTAG
R. ornithinolytica st. HPP19         CACCCCATTA TTTTTGCAAA TATCGGGACT ACCGTTCGCG GAGCTATTGA TAATATTGCC ATCATTCAGC AATCAATTAG
R. ornithinolytica st. HPP15         CACCCCATTA TTTTTGCAAA TATCGGGACT ACCGTTCGCG GAGCTATTGA TAATATTGCC ATCATTCAGC AATCAATTAG
Raoultella planticola st. RP_3045    CACCCCATTA TTTTTGCAAA TATTGGGACT ACCGTTCGCG GAGCAATTGA TAATATTGCC ATCATTCAGG AATCAATTAG
R. planticola st. S8                 CACCCCATTA TTTTTGCAAA TATCGGGACT ACCGTTCGCG GAGCTATTGA TAATATTGCC ATCATTCAGC AATCAATTAG
R. planticola st. Y1-1               CACCCCATTA TTTTTGCAAA TATCGGGACT ACCGTTCGCG GAGCTATTGA TAATATTGCC ATCATTCAGC AATCAATTAG
C. youngae st. NCTC8782              CATCCGATTA TTTTTGCAAA TATCGGCACA ACCGTTCGTG GCGCTATTGA TAATATTGCT GAGATCCAAC AACGGATTGG
C. youngae isolate BB1468            CATCCGATTA TTTTTGCAAA TATCGGCACA ACCGTTCGTG GCGCTATTGA TAATATTGCT GAGATCCAAC AACGGATTGG
PC2 F3                               ---------- ---------- ---------- ---------- ---------- ---------- ---------- ----------
PC2 F1c (r.)                         ---------- ---------- ---------- ---------- ---------- ---------- ---------- ----------
PC2 LF (r.)                          ---------- ---------- ---------- ---------- ---------- ---------- ---------- ----------
PC2 F2                               ---------- ---------- ---------- ---------- ---------- ---------- ---------- ----------
PC2 B3 (r.)                          ---------- ---------- ---------- ---------- ---------- ---------- ---------- ----------
PC2 B1c                              ---------- ---------- ---------- ---------- ---------- ---------- ---------- ----------
PC2 B2 (r.)                          ---------- ---------- ---------- ---------- ---------- ---------- ---------- ----------
PC2 LB                               ---------- ---------- ---------- ---------- ---------- ---------- ---------- ----------
```

```
                                          570        580        590        600        610        620        630        640
K. aerogenes st. Ka37751              TGCGCTGGGG ATCCCACGTG AAGATTATTA TCTACACGCG GATGCCGCGC TAAGCGGCAT GATCTTGCCT TTCGTGGAGG
K. pneumoniae st. GDFK0932            GGCCTATGGT ATCAAGCGAG AGGAATTCTA TCTGCACGCT GACGCCGCAC TGAGTGGTAT GATCCTACCG TTTGTTGACG
K. pneumoniae st. PB270               GGCCTATGGT ATCAAGCGAG AGGAATTCTA TCTGCACGCT GACGCCGCAC TGAGTGGTAT GATCCTACCG TTTGTTGACG
M. morganii st. DI-244                AGCAGCCGGT ATCAAACGTG AAGATTACTA CCTGCACGCT GATGCGGCAC TGAGCGGCAT GATCCTGCCA TTCGTTGATG
M. morganii st. ATCC 9237             AGCAGCCGGT ATCAAACGTG AAGATTACTA CCTGCATGCT GATGCGGCAC TGAGCGGCAT GATCCTGCCA TTCGTTGATG
R. ornithinolytica st. RoM27LC23      CGAACTGGGC ATTGAGCGCA AAGATTATTA TCTTCACGCT GACGCGGCGC TGAGCGGAAT GATCTTACCT TTTGTGGATA
R. ornithinolytica st. HPP19          CGAACTGGGC ATTGAGCGCA AAGATTATTA TCTTCACGCT GACGCGGCGC TGAGCGGAAT GATCTTACCT TTTGTGGATA
R. ornithinolytica st. HPP15          CGAACTGGGC ATTGAGCGCA AAGATTATCA TCTTCACGCT GACGCGGCGC TGAGCGGAAT GATCTTACCT TTTGTGGATA
Raoultella planticola st. RP_3045     CGAACTGGGC ATTGAACGCA AAGATTATTA TCTTCACGCT GACGCGGCGC TGAGCGGAAT GATCTTACCT TTTGTTGATA
R. planticola st. S8                  CGAACTGGGC ATTGAGCGCA AAGATTATTA TCTTCACGCT GACGCGGCGC TGAGTGGAAT GATCTTACCT TTTGTGGATA
R. planticola st. Y1-1                CGAACTGGGC ATTGAGCGCA AAGATTATTA TCTTCACGCT GACGCGGCGC TGAGCGGAAT GATCTTACCT TTTGTGGATA
C. youngae st. NCTC8782               TCAACTGGGT ATCAAACGTG ACGATTATTA TCTACACGCT GATGCTGCGT TGAGCGGTAT GATTTTGCCG TTTGTGAACG
C. youngae isolate BB1468             TCAACTGGGT ATCAAACGTG ACGATTATTA TCTACACGCT GATGCTGCGT TGAGCGGTAT GATTTTGCCG TTTGTGAACG
PC2 F3                                ---------- ---------- ---------- ---------- ---------- ---------- ---------- ----------
PC2 F1c (r.)                          ---------- ---------- ---------- ---------- ---------- ---------- ---------- ----------
PC2 LF (r.)                           ---------- ---------- ---------- ---------- ---------- ---------- ---------- ----------
PC2 F2                                ---------- ---------- ---------- ---------- ---------- ---------- ---------- ----------
PC2 B3 (r.)                           ---------- ---------- ---------- ---------- ---------- ---------- ---------- ----------
PC2 B1c                               ---------- ---------- ---------- ---------- ---------- ---------- ---------- ----------
PC2 B2 (r.)                           ---------- ---------- ---------- ---------- ---------- ---------- ---------- ----------
PC2 LB                                ---------- ---------- ---------- ---------- ---------- ---------- ---------- ----------

                                          650        660        670        680        690        700        710        720
K. aerogenes st. Ka37751              ATCCACAACC GTTTACCTTC GCGGATGGTA TCGATTCGAT TGGCGTTTCC GGACACAAAA TGATCGGCTC TCCGATTCCA
K. pneumoniae st. GDFK0932            AGCCTCAGCC ATTTACTTTC ACAGACGGTA TTGACTCAAT CAGTGTTTCT GGTCACAAAA TGATTGGTTC ACCGATACCT
K. pneumoniae st. PB270               AGCCTCAGCC ATTTACTTTC ACAGACGGTA TTGACTCAAT CAGTGTTTCT GGTCACAAAA TGATTGGTTC ACCG------
M. morganii st. DI-244                ATGCACAGCC ATTCACGTTT GCTGACGGTA TCGACTCAAT CGGTGTTTCC GGCCATAAAA TGATTGGTTC GCCAATCCCT
M. morganii st. ATCC 9237             ATGCACAACC ATTCACGTTT GCTGACGGTA TCGACTCAAT CGGTGTTTCC GGCCATAAAA TGATTGGTTC ACCAATCCCT
R. ornithinolytica st. RoM27LC23      ATCCACAGCC GTTTAACTTC GCAGATGGCA TTGATTCCAT TGGTGTTTCC GGACACAAAA TGATTGGCTC ACCGATTCCT
R. ornithinolytica st. HPP19          ATCCACAGCC GTTTAACTTC GCAGATGGCA TCGATTCTAT TGGTGTTTCC GGACACAAAA TGATTGGCTC ACCGATTCCT
R. ornithinolytica st. HPP15          ATCCACAGCC GTTTAACTTC GCAGATGGCA TTGATTCCAT TGGTGTTTCC GGACACAAAA TGATTGGCTC ACCGATTCCT
Raoultella planticola st. RP_3045     ATCCACAGCC GTTTAACTTC GCAGATGGCA TTGATTCCAT TGGTGTTTCC GGACACAAAA TGATTGGCTC ACCGATTCCT
R. planticola st. S8                  ATCCACAGCC GTTTAACTTC GCAGATGGCA TTGATTCCAT TGGTGTTTCC GGACACAAAA TGATTGGCTC ACCGATTCCT
R. planticola st. Y1-1                ATCCACAGCC GTTTAACTTC GCAGATGGCA TTGATTCCAT TGGTGTTTCC GGACACAAAA TGATTGGCTC ACCGATTCCT
C. youngae st. NCTC8782               ATCCGCAGCC TTTTAACTTC GCTGATGGTA TTGATTCTAT TGGCGTTTCA GGTCACAAAA TGATTGGTTC GCCTATTCCC
C. youngae isolate BB1468             ATCCGCAGCC TTTTAACTTC GCTGATGGTA TTGATTCTAT TGGCGTTTCA GGCCACAAAA TGATTGGTTC GCCTATTCCC
PC2 F3                                ---------- ---------- ---------- ---------- ---------- ---------- ---------- ----------
PC2 F1c (r.)                          ---------- ---------- ---------- ---------- ---------- ---------- ---------- ----------
PC2 LF (r.)                           ---------- ---------- ---------- ---------- ---------- ---------- ---------- ----------
PC2 F2                                ---------- ---------- ---------- ---------- ---------- ---------- ---------- ----------
PC2 B3 (r.)                           ---------- ---------- ---------- ---------- ---------- ---------- ---------- ----------
PC2 B1c                               ---------- ---------- ---------- ---------- ---------- ---------- ---------- ----------
PC2 B2 (r.)                           ---------- ---------- ---------- ---------- ---------- ---------- ---------- ----------
PC2 LB                                ---------- ---------- ---------- ---------- ---------- ---------- ---------- ----------

                                          730        740        750        760        770        780        790        800
K. aerogenes st. Ka37751              TGCGGCATTG TGGTAGCCAA GAAAGCCAAC GTCGACCGTA TCAGCGTAGA GATCGACTAC ATCTCCGCCC ACGATAAAAC
K. pneumoniae st. GDFK0932            TGTGGTATCG TACTGGCGAA GAAAGATCAT GTAAATCGTA TTTCAGTAGC TGTAGATTAC ATTTCAACTC ATGATAAGAC
K. pneumoniae st. PB270               ---------- ---------- ---------- ---------- ---------- ---------- ---------- ----------
M. morganii st. DI-244                TGCGGTATCG TTGTTGCGAA GAAAGAAAAC GTGGATCGTA TTTCTGTTGA AATCGACTAC ATCTCCGCAC ACGACAAAAC
M. morganii st. ATCC 9237             TGCGGTATCG TTGTTGCGAA GAAAGAAAAC GTGGATCGTA TTTCTGTTGA AATCGACTAC ATCTCCGCAC ACGACAAAAC
R. ornithinolytica st. RoM27LC23      TGCGGCATTG TTGTTGCTAA AAAGAAAAAT GTCGATCGGA TCTCTGTAGA GATTGATTAT ATTTCTGCTC ACGATAAAAC
R. ornithinolytica st. HPP19          TGCGGCATTG TTGTTGCTAA AAAGAAAAAT GTCGATCGGA TCTCTGTAGA GATTGATTAT ATTTCTGCTC ACGATAAAAC
R. ornithinolytica st. HPP15          TGCGGCATTG TTGTTGCTAA AAAGAAAAAT GTCGATCGGA TCTCTGTAGA GATTGATTAT ATTTCTGCTC ACGATAAAAC
Raoultella planticola st. RP_3045     TGCGGCATTG TTGTTGCTAA AAAGAAAAAT GTCGATCGAA TCTCTGTAGA GATTGATTAT ATTTCTGCTC ACGATAAAAC
R. planticola st. S8                  TGCGGCATTG TTGTTGCTAA AAAGAAAAAT GTCGATCGGA TCTCTGTAGA GATTGATTAT ATTTCTGCTC ACGATAAAAC
R. planticola st. Y1-1                TGCGGCATTG TTGTTGCTAA AAAGAAAAAT GTCGATCGGA TCTCTGTAGA GATTGATTAT ATTTCTGCTC ACGATAAAAC
C. youngae st. NCTC8782               TGCGGCATCG TGGTAGCGAA AAGGAAAAAC GTTGATCGGA TTTCTGTCGA AATTGATTAC ATCTCTGCAC ACGATAAAAC
C. youngae isolate BB1468             TGCGGCATCG TGGTAGCGAA AAGGAAAAAC GTTGATCGGA TTTCTGTCGA AATTGATTAC ATCTCTGCAC ACGATAAAAC
PC2 F3                                ---------- ---------- ---------- ---------- ---------- ---------- ---------- ----------
PC2 F1c (r.)                          ---------- ---------- ---------- ---------- ---------- ---------- ---------- ----------
PC2 LF (r.)                           ---------- ---------- ---------- ---------- ---------- ---------- ---------- ----------
PC2 F2                                ---------- ---------- ---------- ---------- ---------- ---------- ---------- ----------
PC2 B3 (r.)                           ---------- ---------- ---------- ---------- ---------- ---------- ---------- ----------
PC2 B1c                               ---------- ---------- ---------- ---------- ---------- ---------- ---------- ----------
PC2 B2 (r.)                           ---------- ---------- ---------- ---------- ---------- ---------- ---------- ----------
PC2 LB                                ---------- ---------- ---------- ---------- ---------- ---------- ---------- ----------

                                          810        820        830        840        850        860        870        880
K. aerogenes st. Ka37751              GATTTCAGGG TCACGCAACG GTCATACCCC TTTGATGATG TGGGCCGCCG TTCGCAGCCA TACCGATGCC GAATGGCATC
K. pneumoniae st. GDFK0932            TATTACAGGC TCACGCAATG GCCATACACC GTTGATTATG TGGGAAGCCA TTCGCAGTCA CTCCTATTCC GACTGGAAAC
K. pneumoniae st. PB270               ---------- ---------- ---------- ---------- ---------- ---------- ---------- ----------
M. morganii st. DI-244                CATCACCGGT TCACGTAACG GTCACACACC ACTGATGCTG TGGGAAGCTA TCCGTTCACA TTCAACTGAG GAATGGAAAC
M. morganii st. ATCC 9237             CATCACCGGT TCACGTAACG GTCACACACC AGTGATGCTG TGGGAAGCTA TCCGTTCACA TTCAGCTGAG GAATGGAAGC
R. ornithinolytica st. RoM27LC23      CATTTCAGGT TCGCGTAACG GTCATACCCC ACTGATGATG TGGGAGGCAA TTCGTAGCCA TTCATGGGAG GAGTGGCGGC
R. ornithinolytica st. HPP19          CATTTCAGGT TCGCGTAACG GCCACACCCC TCTCATGCTG TGGG------ ---------- ---------- ----------
R. ornithinolytica st. HPP15          CATTTCAGGT TCGCGTAACG GCCACACTCC ACTCATGCTG TGGG------ ---------- ---------- ----------
Raoultella planticola st. RP_3045     CATTTCAGGC TCGCGTAACG GTCATACCCC ACTGATGATG TGGGAGGCAA TTCGTAGCCA TTCATGGGAG GAGTGGCGGC
R. planticola st. S8                  CATTTCAGGT TCGCGTAACG GTCATACCCC ACTCATGATG TGGGAGGCAA TTCGTAGCCA TTCATGGGAG GAGTGGCGGC
R. planticola st. Y1-1                CATTTCAGGT TCGCGTAACG GTCATACCCC ACTCATGATG TGGGAGGCAA TTCGTAGCCA TTCATGGGAG GAGTGGCGGC
C. youngae st. NCTC8782               CATTTCTGGT TCTCGTAACG GCCATACACC GCTAATGATG TGGGAAGCTA TTCGCAGCCA TTCCTGGAGC GACTGGCAAC
C. youngae isolate BB1468             CATTTCTGGT TCTCGTAATG GCCATACACC GCTAATGATG TGGGAAGCTA TTCGCAGCCA TTCCTGGAGC GACTGGCAAC
PC2 F3                                ---------- -------ACG GTCATACCCC TTTGATG--- ---------- ---------- ---------- ----------
PC2 F1c (r.)                          ---------- ---------- ---------- ---------- ---------- ---------- ---------- ----------
PC2 LF (r.)                           ---------- ---------- ---------- ---------- ---------- ---------- ---------- ----------
PC2 F2                                ---------- ---------- ---------- ---------- ---------G TTCGCAGCCA TACCGAT--- ----------
PC2 B3 (r.)                           ---------- ---------- ---------- ---------- ---------- ---------- ---------- ----------
PC2 B1c                               ---------- ---------- ---------- ---------- ---------- ---------- ---------- ----------
PC2 B2 (r.)                           ---------- ---------- ---------- ---------- ---------- ---------- ---------- ----------
PC2 LB                                ---------- ---------- ---------- ---------- ---------- ---------- ---------- ----------
```

```
                                            890        900        910        920        930        940        950        960
K. aerogenes st. Ka37751               GCCGCATTGG TCACAGCCTC AACATGGCGA AATACGCCGT GGATCGCTTT AAAGCAGCAG GTATTGACGC GCTGTGCCAC
K. pneumoniae st. GDFK0932             AACGTATAGA GCATTGTATG AATTTGGCAC AATACGTTGT TGATAATTTC CAGAAAGCTG GCATTAATGC TTGGCGCAAC
K. pneumoniae st. PB270                ---------- ---------- ---------- ---------- ---------- ---------- ---------- ----------
M. morganii st. DI-244                 GCCGCATCAC CCGCAGTCTG GATATGGCTC AGTATGCTGT TGACCGTATG CAGAAAGCCG GTATCAATGC ATGGCGCAAC
M. morganii st. ATCC 9237              GCCGCATCAC CCGCAGTCTG GATATGGCTC AGTACGCTGT TGACCGTATG CAGAAAGCCG GTATCAATGC ATGGCGCAAC
R. ornithinolytica st. RoM27LC23       GTCGCATCGA ACGCAGCCTG AATATGGCGC AATATGCCGT TGACCGTTTT CAGTCCGCGG GAATTGATGC CTGGCGCAAC
R. ornithinolytica st. HPP19           ---------- ---------- ---------- ---------- ---------- ---------- ---------- ----------
R. ornithinolytica st. HPP15           ---------- ---------- ---------- ---------- ---------- ---------- ---------- ----------
Raoultella planticola st. RP_3045      GTCGTATCGA ACGCAGCCTG AATATGGCGC AATATGCCGT TGACCGTTTT CAGTCCGCGG GAATTGATGC CTGGCGCAAC
R. planticola st. S8                   GTCGCATCGA ACGCAGCCTG AATATGGCGC AATATGCCGT TGACCGTTTT CAGTCCGCGG GAATTGATGC CTGGC-----
R. planticola st. Y1-1                 GTCGCATCGA ACGCAGCCTG AATATGGCGC AATATGCCGT TGACCGTTTT CAGTCCGCGG GAATTGATGC CTGGC-----
C. youngae st. NCTC8782                GCCGCATTGA ACATAGCCTG AATATGGCGC AGTACGCAGT CGATCGCTTA CAGGCTGCTG GTATTGATGC CTGGCGCAAT
C. youngae isolate BB1468              GCCGCATTGA ACATAGCCTG AATATGGCGC AGTACGCAGT CGATCGCTTA CAGGCTGCTG GTATTGATGC CTGGCGCAAT
PC2 F3                                 ---------- ---------- ---------- ---------- ---------- ---------- ---------- ----------
PC2 F1c (r.)                           ---------- -------CTC AACATGGCGA AATACGCCG- ---------- ---------- ---------- ----------
PC2 LF (r.)                            GCCGCATTGG TCACAGC--- ---------- ---------- ---------- ---------- ---------- ----------
PC2 F2                                 ---------- ---------- ---------- ---------- ---------- ---------- ---------- ----------
PC2 B3 (r.)                            ---------- ---------- ---------- ---------- ---------- ---------- ---------- ----------
PC2 B1c                                ---------- ---------- ---------- ---------- ---------- -AAGCAGCAG GTATTGACGC GC--------
PC2 B2 (r.)                            ---------- ---------- ---------- ---------- ---------- ---------- ---------- ----------
PC2 LB                                 ---------- ---------- ---------- ---------- ---------- ---------- ---------- -------CAC

                                            970        980        990       1000       1010       1020       1030       1040
K. aerogenes st. Ka37751               AAAAACTCCA TCACGGTGGT CTTTCCGAAG CCTTCTGAAT GGGTATGGAA GAAACACTGC CTGGCAACGT CCGGCAACGT
K. pneumoniae st. GDFK0932             AAAAACTCAA TTACAGTGAT CTTTCCCTGC CCCTCTGAAT CTGTATGGAA GAAACACGGC TTAGCGATCT CTGGCAATAT
K. pneumoniae st. PB270                ---------- ---------- ---------- ---------- ---------- ---------- ---------- ----------
M. morganii st. DI-244                 AAAAACTCCA TCACTGTTGT GTTCCCTTGC CCGTCAGAAC GCGTCTGGAG AGAACATTGC CTGGCAACGT CCGGCGACGT
M. morganii st. ATCC 9237              AAGAACTCCA TCACTGTTGT GTTCCCTTGC CCGTCAGAAC GCGTCTGGAG AGAACATTGC CTGGCAACTT CCGGCGACGT
R. ornithinolytica st. RoM27LC23       AAAAACTCCA TCACCGTCGT CTTCCCTTGC CCATCAGAGG CCGTCTGGAA GAAACACTGT CTTGCTACAT CTGGCGATAT
R. ornithinolytica st. HPP19           ---------- ---------- ---------- ---------- ---------- ---------- ---------- ----------
R. ornithinolytica st. HPP15           ---------- ---------- ---------- ---------- ---------- ---------- ---------- ----------
Raoultella planticola st. RP_3045      AAAAACTCCA TCACCGTCGT CTTCCCTTGC CCATCAGAGG CCGTCTGGAA GAAACACTGT CTTGCTACAT CTGGCGATAT
R. planticola st. S8                   ---------- ---------- ---------- ---------- ---------- ---------- ---------- ----------
R. planticola st. Y1-1                 ---------- ---------- ---------- ---------- ---------- ---------- ---------- ----------
C. youngae st. NCTC8782                AAAAACTCTA TTACCGTGGT TTTCCCTTGT CCGTCAGAAG CTGTCTGGAA GAAGCACTGT CTGGCCACCT CCGGTGACAT
C. youngae isolate BB1468              AAAAACTCTA TTACCGTGGT TTTCCCTTGT CCGTCAGAAG CTGTCTGGAA GAAGCACTGT CTGGCCACCT CCGGTGACAT
PC2 F3                                 ---------- ---------- ---------- ---------- ---------- ---------- ---------- ----------
PC2 F1c (r.)                           ---------- ---------- ---------- ---------- ---------- ---------- ---------- ----------
PC2 LF (r.)                            ---------- ---------- ---------- ---------- ---------- ---------- ---------- ----------
PC2 F2                                 ---------- ---------- ---------- ---------- ---------- ---------- ---------- ----------
PC2 B3 (r.)                            ---------- ---------- ---------- ---------- ----ATGGAA GAAACACTGC CTGG------ ----------
PC2 B1c                                ---------- ---------- ---------- ---------- ---------- ---------- ---------- ----------
PC2 B2 (r.)                            ---------- ---------- ----CCGAAG CCTTCTGAAT GGG------- ---------- ---------- ----------
PC2 LB                                 AAAAACTCCA TCACGGTGG- ---------- ---------- ---------- ---------- ---------- ----------

                                           1050       1060       1070       1080       1090       1100       1110       1120
K. aerogenes st. Ka37751               AGCCCATCTG ATCACCACCG CTCACCACCT GGACAGTTCC CGGATTGATG CGCTGATCGA TGACGTAATT GCCGACCTGG
K. pneumoniae st. GDFK0932             CGCTCACCTA ATCACTACAC CGCATCATTT TGATAGTAGT AAAATAGACT CATTGATCAT TGACGTTATT GCCGATCTGA
K. pneumoniae st. PB270                ---------- ---------- ---------- ---------- ---------- ---------- ---------- ----------
M. morganii st. DI-244                 GGCTCACCTG ATCACCACCG CGCACCATCT GGATACCGCG CAGATCGACA AACTGATCGA CGACGTTATC GCGGATTTTA
M. morganii st. ATCC 9237              GGCTCACCTG ATTACCACCG CGCACCATCT GGATACCGCT CAGATCGACA AACTGATCGA CGACGTTATC GCGGATTTTA
R. ornithinolytica st. RoM27LC23       TGCCCACCTC ATCGCCACCG CGCACCACCT TGATTCCAGC AAAATTGATG CACTGATTGA TGACGTGATC GCCGACCTGA
R. ornithinolytica st. HPP19           ---------- ---------- ---------- ---------- ---------- ---------- ---------- ----------
R. ornithinolytica st. HPP15           ---------- ---------- ---------- ---------- ---------- ---------- ---------- ----------
Raoultella planticola st. RP_3045      TGCCCACCTC ATCGCCACCG CGCACCACCT TGATTCCAGC AAAATTGATG CACTGATTGA TGACGTGATC GCCGACCTGA
R. planticola st. S8                   ---------- ---------- ---------- ---------- ---------- ---------- ---------- ----------
R. planticola st. Y1-1                 ---------- ---------- ---------- ---------- ---------- ---------- ---------- ----------
C. youngae st. NCTC8782                CGCTCACCTG ATTACCACTG CGCACCACCT TGACTCCAGC AAGATTGATG AGCTGATTGA CGATGTGATT GCTGACCTGA
C. youngae isolate BB1468              CGCTCACCTG ATTACCACTG CGCACCACCT TGACTCCAGC AAGATTGATG AGCTGATTGA CGATGTGATT GCTGACCTGA
PC2 F3                                 ---------- ---------- ---------- ---------- ---------- ---------- ---------- ----------
PC2 F1c (r.)                           ---------- ---------- ---------- ---------- ---------- ---------- ---------- ----------
PC2 LF (r.)                            ---------- ---------- ---------- ---------- ---------- ---------- ---------- ----------
PC2 F2                                 ---------- ---------- ---------- ---------- ---------- ---------- ---------- ----------
PC2 B3 (r.)                            ---------- ---------- ---------- ---------- ---------- ---------- ---------- ----------
PC2 B1c                                ---------- ---------- ---------- ---------- ---------- ---------- ---------- ----------
PC2 B2 (r.)                            ---------- ---------- ---------- ---------- ---------- ---------- ---------- ----------
PC2 LB                                 ---------- ---------- ---------- ---------- ---------- ---------- ---------- ----------
```

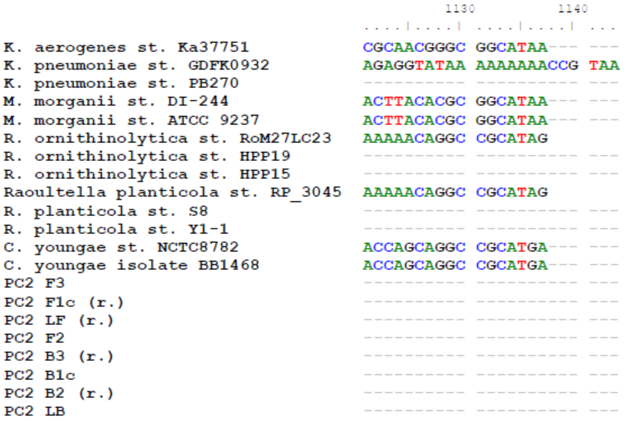

Supplementary Data S2. Sequence alignment of the LAMP assay target region of *Klebsiella aerogenes HDC* gene with *HDC* gene sequences from *HDC*-producing *Enterobacteriaceae*—*Klebsiella pneumoniae*, *Morganella morganii*, *Raoultella ornithinolytica*, *Raoultella planticola* and *Citrobacter youngae* (Table S4). Sequence alignment was performed using BioEdit v7.2.0 software (Informer Technologies, Inc.).

a

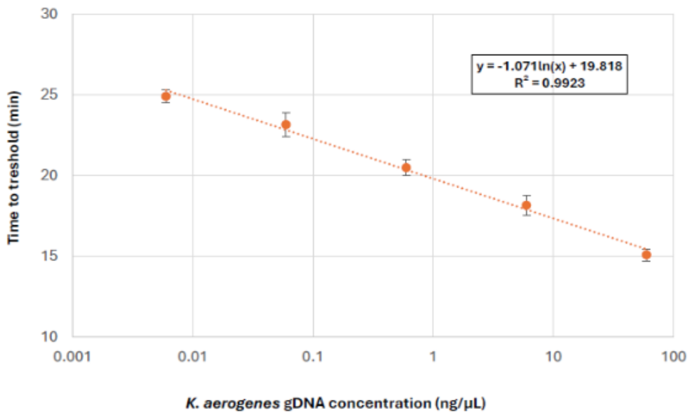

b

K. aerogenes gDNA concentration (ng/µL)	Tt average (min)	SD (min)	CV (%)
59.9	15.08	0.38	2.53
5.99	18.17	0.63	3.46
0.599	20.50	0.50	2.44
0.0599	23.17	0.76	3.30
0.00599	24.92	0.38	1.53

Supplementary Data S3. Linear range analysis and variability of the LAMP assay for *Klebsiella aerogenes* gDNA. (a) Standard curve illustrating the relationship between time-to-threshold (Tt) and *K. aerogenes* gDNA concentration used to assess the linear detection range of the LAMP assay. (b) Table summarizing the coefficient of variation (CV) of Tt values calculated from replicate reactions during the LoD determination experiment.
